# A Deep Generative Model for the Inverse Design of Transition Metal Ligands and Complexes

**DOI:** 10.1021/jacsau.5c00242

**Published:** 2025-04-23

**Authors:** Magnus Strandgaard, Trond Linjordet, Hannes Kneiding, Arron L. Burnage, Ainara Nova, Jan Halborg Jensen, David Balcells

**Affiliations:** † Hylleraas Centre for Quantum Molecular Sciences, Department of Chemistry, University of Oslo, P.O. Box 1033, Blindern, Oslo 0315, Norway; ‡ Department of Chemistry, University of Copenhagen, Copenhagen 2100, Denmark; § Centre for Materials Science and Nanotechnology, Department of Chemistry, University of Oslo, Oslo N-0315, Norway

**Keywords:** inverse design, transition metal complex, metal ligand, SMILES, variational autoencoder, density functional theory, conditional generation, tmQM data sets

## Abstract

Deep generative models yielding transition metal complexes (TMCs) remain scarce despite the key role of these compounds in industrial catalytic processes, anticancer therapies, and the energy transition. Compared to drug discovery within the chemical space of organic molecules, TMCs pose further challenges, including the encoding of chemical bonds of higher complexity and the need to optimize multiple properties. In this work, we developed a generative model for the inverse design of transition metal ligands and complexes, based on the junction tree variational autoencoder (JT-VAE). After implementing a SMILES-based encoding of the metal–ligand bonds, the model was trained with the tmQMg-L ligand library, allowing for the generation of thousands of novel, highly diverse monodentate (κ^1^) and bidentate (κ^2^) ligands, including imines, phosphines, and carbenes. Further, the generated ligands were labeled with two target properties reflecting the stability and electron density of the associated homoleptic iridium TMCs: the HOMO–LUMO gap (ϵ) and the charge of the metal center (*q*_Ir_). This data was used to implement a conditional model that generated ligands from a prompt, with the single- or dual-objective of optimizing either or both the ϵ and *q*_Ir_ properties and allowing for chemical interpretation based on the optimization trajectories. The optimizations also had an impact on other chemical properties, including ligand dissociation energies and oxidative addition barriers. A similar model was implemented to condition ligand generation by solubility and steric bulk.

## Graphical abstract

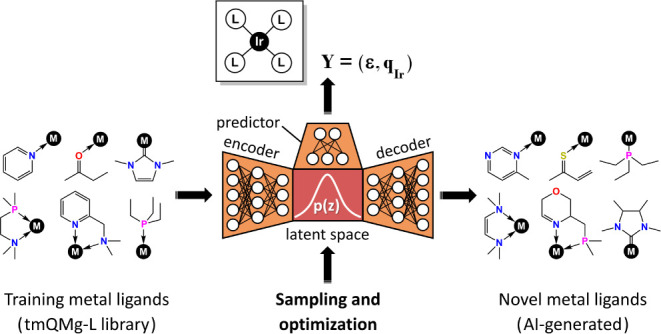

## Introduction

Transition metal complexes (TMCs) are at the core of highly relevant chemical technologies, with examples including catalysis^1–3^ and chemotherapy.^4–6^ Socioeconomic issues like the energy crisis and the green shift are related to TMCs, which can enable key processes like the sustainable generation and use of hydrogen.^7–9^ Hence, there is a need for accelerating TMC discovery, a challenge in which machine learning (ML) methods are playing an increasingly relevant role.^10–13^ ML can be used to optimize accurate surrogate models of computationally demanding methods like DFT,^14–17^ enabling discovery funnels in the exploration of vast chemical spaces.^18–22^ Predictive ML models have already proven successful in challenging tasks over a wide range of applications, including catalysis,^23–27^ medicinal chemistry,^28–31^ and materials science.^32–36^

Generative ML^37–41^ is a compelling complement to predictive ML. This approach aims at leveraging data to make more data of additional value. Popular examples include image and text generation with DALL-E^42^ and ChatGPT^43^ AIs, respectively, with large language models emerging as a valuable research tool in chemistry.^44–51^ The fundamental idea is to reverse the mapping of a system representation (*X*) to a property of interest (*y*) from *X* → *y* (prediction) to *X* ← *y* (generation), enabling inverse design tasks in which a target y* value can be used to condition the generation of X* molecules or materials for which *y* ≈ y*.^52–55^ An advantage of generative ML is its ability to explore chemical spaces without enumerating them from predefined structural templates.

Several generative methods are currently available for molecules and materials, including reinforcement^56,57^ and active^58^ learning, diffusion and flow models,^59–66^ generative adversarial networks,^67,68^ and autoencoders.^69,70^ Interestingly, genetic algorithms may not fit into the somehow blurred definition of generative ML and yet they have achieved top performance in several design tasks when the system of interest has a modular nature codable as a chromosome, like TMCs,^71–77^ though the cost of the fitness can be a significant limiting factor.^78^

Variational autoencoders (VAEs)^79^ are a powerful approach to inverse design. In general, VAEs do the task *X* ⇒ *z* ⇒ *X*, where ⇒ can be a graph neural network if *X* is a graph, and *z* is the latent space, an internal representation learned as a probability distribution, *p*(*z*). The latent space can be seen as an encoding of *X* and, therefore, the *X* ⇒ *z* and *z* ⇒ *X* models act as an encoder and a decoder, respectively. VAEs are trained by minimizing a loss function that accounts for the reconstruction error (the difference between the encoded and decoded *X*) and a regularization term (the difference between the *p*(*z*) and normal distributions). Further, VAEs allow for jointly training a differentiable *z* → *y* mapping for conditional generation, in which X* can be decoded from a z* value minimizing (or maximizing) *y* to a y* target value. Figure 1A illustrates these concepts for the VAE architecture developed in this work.

**Figure 1:**
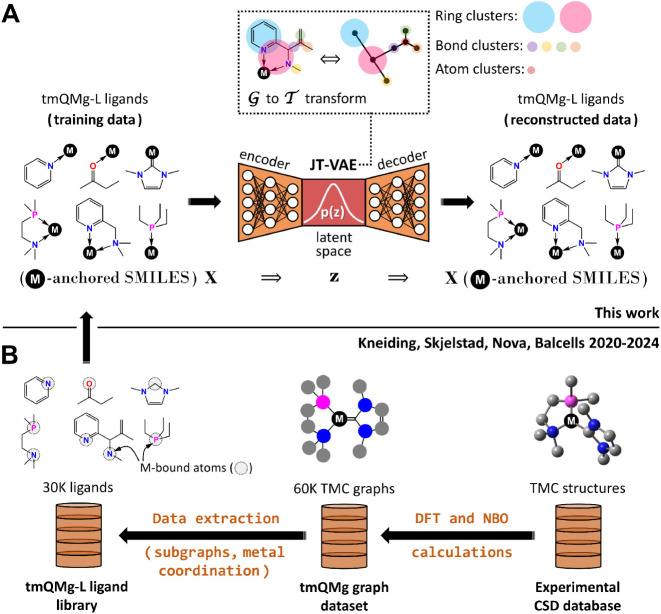
Generation of metal ligands: (A) JT-VAE model, including the transformation from graphs ( G ) to junction trees ( T ), operating on a SMILES-based encoding; (B) Derivation of the training data from the CSD database through the computational tmQMg and tmQMg-L data sets; M = Metal.

Compared to organic medicinal chemistry, the use of VAEs and other generative deep learning methods in TMC discovery has only a few precedents. Schilter et al. reported a VAE for the inverse design of cross-coupling TMC catalysts, which generated 8.6K candidates.^80^ Yang et al.^81^ developed another VAE model generating TMCs with optimal energy gaps between low- and high-spin states. More recently, Jin and Merz reported a diffusion model for ligand generation over the open coordination sites of predefined scaffolds, yielding TMCs with high synthetic accessibility scores.^82,83^ Bhowmik implemented a similar model, OM-DIFF,^84^ adding equivariance and testing its performance in the same inverse design problem addressed by Schilter et al.^80^ All models achieved high levels of validity, uniqueness, and novelty in their generated output. However, the conditioning of the generative tasks by more than one property was not investigated in all cases. Other drawbacks, differing by the model, were the lack of either explicit metal–ligand bonds or H atoms in the representation, and the use of training data sets with limited diversity.

We hereby present an inverse design model that enables the optimization of one or two target properties through the generation of diverse metal ligands, including imines, phosphines, and carbenes, among other types. The model encodes metal–ligand bonds and H atoms explicitly using the junction tree variational autoencoder (JT-VAE) architecture,^85^ (Figure 1A) which was trained with the tmQMg-L data set,^77^ a synthesizable and large 30k ligand library derived from real TMCs in the experimental Cambridge Structural Database (CSD; Figure 1B).^86^ We also developed a SMILES-based string representation of the ligand coordination, which, for neutral dentic κ^*n*^-ligands, allows for encoding and decoding the denticity order *n* and the formal metal–ligand bond order. The JT-VAE model transforms these strings into molecular graphs that are coarse-grained into junction trees of atomic clusters, enforcing chemical validity with a fragment-by-fragment generation process that is more robust than atom-by-atom approaches.

The JT-VAE model can be optimized for unconditional generation, yielding thousands of novel, unique, and valid ligands, without any explicit bias (Figure 2). For conditional generation, these ligands are first used to assemble homoleptic TMCs for which the geometry and other quantum properties are computed with a DFT method to define a set of target properties (*Y*). The conditional model is then optimized after adding a neural network that predicts the *Y* properties from the latent space. This model takes ligands as input prompts and, after the optimization of Y along a user-selected direction, it decodes the associated latent representation into the corresponding optimal ligand. This approach was benchmarked for homoleptic [IrL_4_]^+^ (κ^1^-L) and [IrL_2_]^+^ (κ^2^-L) TMCs, which were inversely designed by optimizing their HOMO–LUMO gap (ϵ), reflecting stability, and the atomic charge of iridium (*q*_Ir_), reflecting the electron density of the metal center. A similar scheme was used to implement a conditional model for the generation of free ligands with the desired steric bulk and solubility.

**Figure 2:**
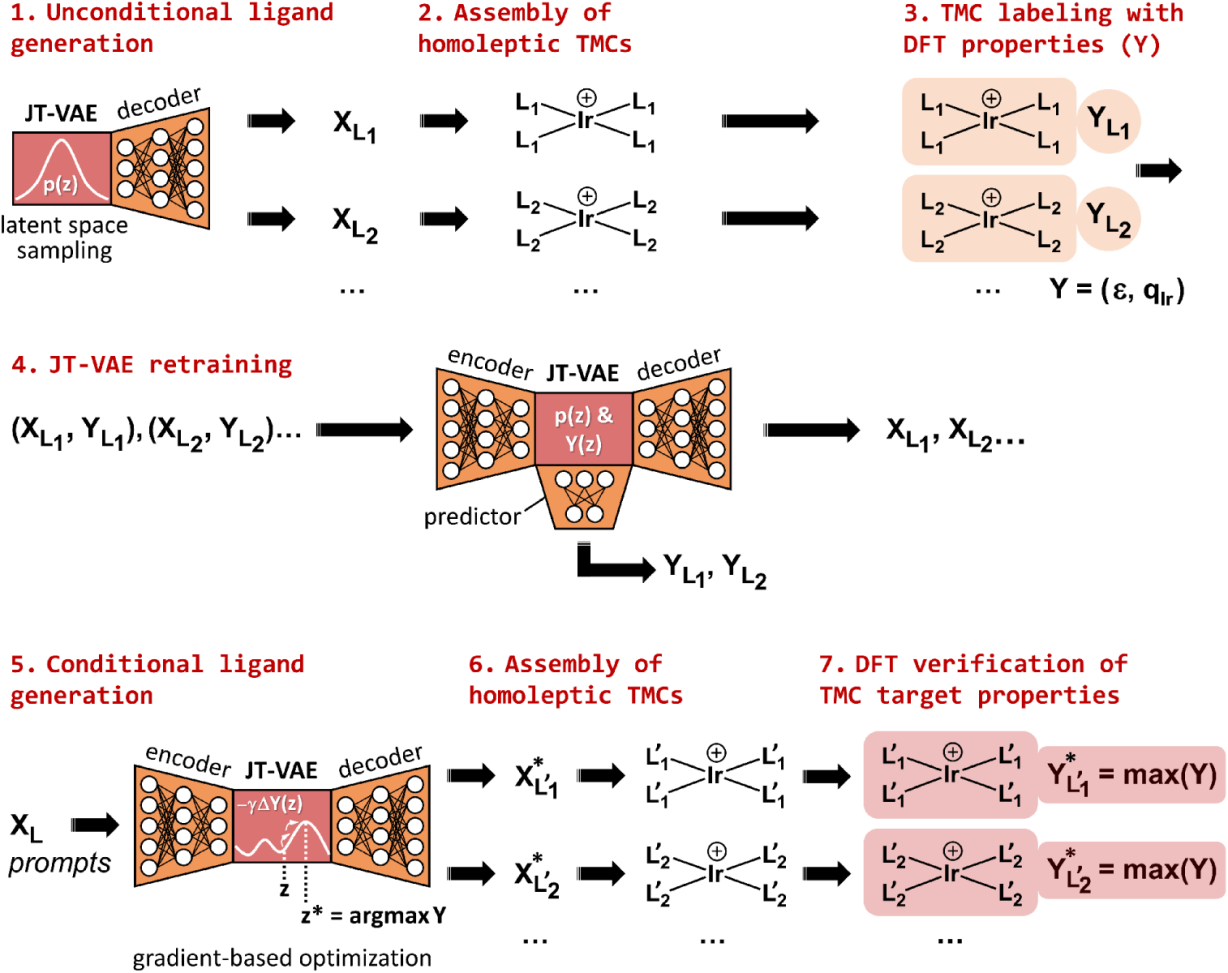
Two-objective inverse design, including the unconditional generation of metal ligands by a JT-VAE model, followed by retraining with the DFT properties *Y* = (ϵ, *q*_Ir_) and the conditional generation of homoleptic TMCs with target properties *Y* = Y*. In the example, Y* is a maximum of *Y* but it can also be a minimum. ϵ = HOMO–LUMO gap, *q*_Ir_ = local charge on iridium.

## Methods

The string representations were developed using the *xyz2mol*, *OpenBabel*, and *RDKit* programs. All TMCs’ DFT data was computed with ORCA^87^ at the DFT levels PBE-D4/def2SVP^88–90^ (optimized geometries) and PBE0-D4/def2TZVP^89–91^ (single point energies) on structures assembled with *molSimplify*.^92^ The free ligands log *P* data was computed with *RDKit*, and the Bk_M_ steric parameter was computed as described in the Supporting Information, which provides further details on the methods used.

### Metal–Ligand Representation

A generative model for TMCs should yield ligands with a valid metal coordination mode. In principle, any molecule can be formulated as a ligand by defining metal anchors after the generation task. However, this approach would not leverage the metal coordination data of the tmQMg-L library, which is highly valuable since it relates directly to the experimental information on the CSD database. We thus decided to keep and express this information in a way compatible with the JT-VAE, allowing for both its encoding and decoding at the input and output levels, respectively.

We combined the *xyz2mol*, *OpenBabel*, and *RDKit* programs to transform the Cartesian coordinates of the tmQMg-L ligands into a SMILES string representing the metal coordination mode (Figure 3). This SMILES expressed the connectivity to the metal center ([M]) with either chains (κ^1^-coordination mode) or rings (κ^2^-coordination mode, yielding metallacycles). For example, the κ^1^-coordination mode of ethylenediamine would be encoded as NCCN­[M], whereas the κ^2^-coordination form would be C1N­[M]­NC1. In these strings, [M] should be somehow expressed without using any of the elements that are commonly found in organic molecules, which, in the SMILES notation system, are subject to valence rules that do not apply to the transition metals.

**Figure 3:**
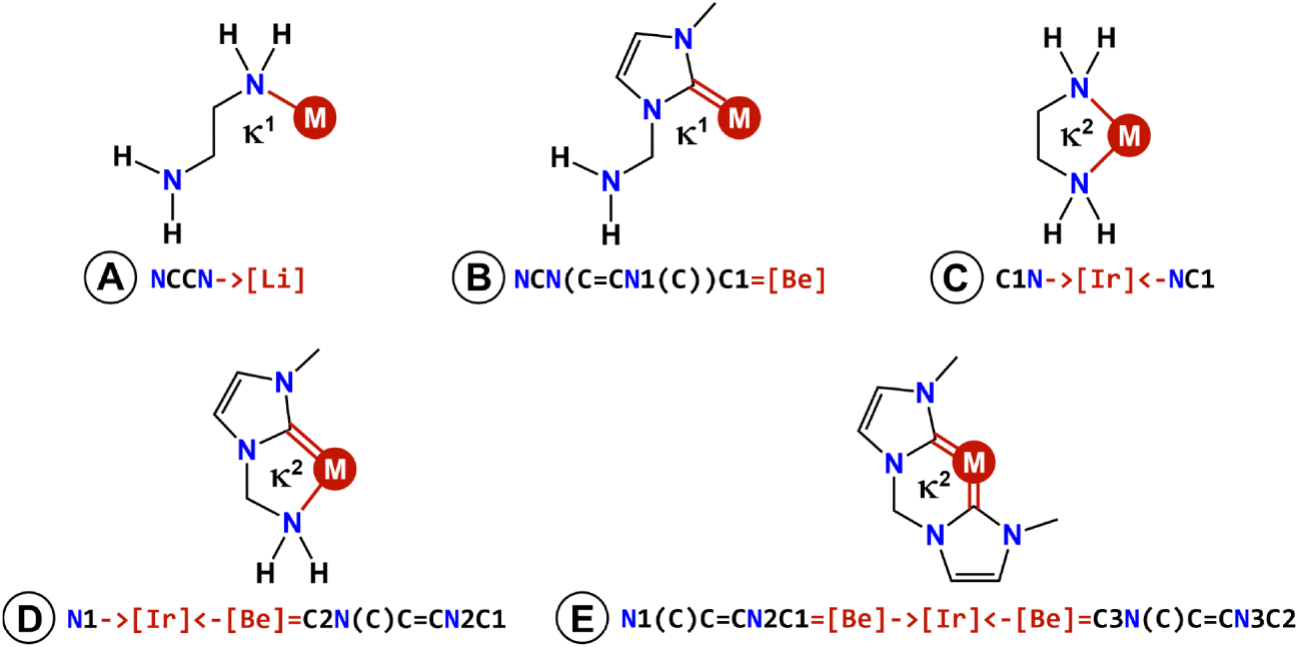
SMILES representations of the κ^1^ (A and B) and κ^2^ (C–E) coordination modes used to train the JT-VAE models of this work. Metal–NHC bonds are drawn as formally double though they may also appear in the literature as single, when π-backdonation is weak. The metal atom, which is represented by the [Li], [Be], and [Ir] encodings, can be any transition metal.

For κ^1^-bound ligands, L–M bonds with a formal bond order equal to one were expressed as L→[Li], where L can be for example an amine or a phosphine. The dative bond, →, is compatible with the SMILES valence rules, which, in addition, support lithium as a monovalent atom, using the [Li] encoding. Since this element is not present in the tmQMg-L training data, the encoding did not introduce any spurious redundancy. The final SMILES string of κ^1^-ethylenediamine used to train the JT-VAE was thus NCCN→[Li] (example A in Figure 3). In contrast, L–M bonds with a formal bond order equal to two were expressed as L=[Be], where L can be a carbene or silylene ligand (example B in Figure 3). In this case, beryllium was used as a bivalent atom, which, with the SMILES [Be] notation, supports the double bond binding without being present in the tmQMg-L training data.

For κ^2^-bound ligands, whereas the same → and = bond strings were used, the metal was encoded as [Ir] to support valencies higher than two. For single bonds, the encoding was L→[Ir]←L′, where L and L′ form a metallacycle; for example, the SMILES of κ^2^-ethylenediamine was C1N→[Ir]←NC1 (C in Figure 3). When there were bonds with a formal order equal to two, the representations became more complex since the =[Be] substring had to be kept to avoid valence errors. The resulting encodings were L→[Ir]←[Be]=L′ and L=[Be]→[Ir]←[Be]=L′ (D and E in in Figure 3). In these cases there are two or three [M] encodings, which nevertheless represent the ligand coordination to a single metal center.

Using these encodings, the SMILES generated by the JT-VAE could be directly interpreted as metal ligands with a well-defined coordination mode. The coordination centers represented by the [Li], [Be], and [Ir] strings, can be replaced by any transition metal. All metal–ligand bond encodings are automated by the code provided. The choice of Li, Be, and Ir was arbitrary and other elements may have served the same purpose. SMILES canonicalization was implemented within the model using RDKit.

### Unconditional Ligand Generation

We trained two separate JT-VAE models: one for the generation of κ^1^ ligands and one for the generation of κ^2^ ligands. Neutral ligands with a minimum of 4 heavy atoms for which the metal is bound to either one or two nonadjacent atoms were used to train the κ^1^ and κ^2^ models, respectively. The training sets contained 4511 κ^1^ and 4582 κ^2^ ligands, all extracted from the tmQMg-L library,^77^ which is part of the tmQM data set series, currently including DFT data at the TPSSh,^93^ PBE(0),^94^ and ωB97M-V^95^ levels of theory.

The use of the SMILES notation for the derivation of the junction trees in the JT-VAE (Figure 1A) was problematic for some ligands. For example: the saturation of the low-valent C atoms of carbenes with two H atoms satisfied the octet rule, but the subsequent addition of a dative bond to encode metal coordination yielded pentavalent C atoms in some generated ligands. The combination of the L=Be encoding (Figure 3) with the corresponding resonance form (Figure S1) solved this issue. We also observed the generation of ligands with high-valent anionic atoms for elements like P, which originated from training ligands that were assigned erroneous charges. These ligands were identified and removed from the training data set to guarantee the chemical validity of the output.

The JT-VAE models were trained and tested in the generation of two 50k ligand sets, one being κ^1^ and the other κ^2^. This generation task was unconditional; that is, the latent space was randomly sampled, without any explicit bias. Table 1 shows the five criteria used to assess the quality of the JT-VAE output. The first is the percentage of ligands that were generated with a valid metal coordination mode. The model was very robust in this regard, with 100% (κ^1^) and 96% (κ^2^) of the ligands containing the appropriate number of metal anchors from the {[Li], [Be], [Ir]} set (Figure 3). For the valid ligands, we next assessed their uniqueness (that is, not repeating) and, for the unique, their novelty relative to the training data. The quality in terms of these criteria was moderate for the uniqueness, 69% (κ^1^) and 59% (κ^2^), and high for the novelty, 95% (κ^1^) and 93% (κ^2^).

**Table 1:** Quality Metrics of the Unconditional Ligand Generation Tasks with the κ^1^ and κ^2^ JT-VAE Models

Set	κ^*n*^	Valid^a^ →	Unique^b^ →	Novel^c^	Diversity^d^ ^e^	Synthesizability^e^ ^f^
Training	*n* = 1	100%	100%	—	0.07, 0.87	0.53, 51%
Training	*n* = 2	100%	100%	—	0.08, 0.85	0.59, 59%
Training	*n* = 1,2	100%	100%	—	0.07, 0.88	0.56, 55%
Generated	*n* = 1	100%	69%	95%	0.08, 0.87	0.45, 44%
Generated	*n* = 2	96%	59%	93%	0.07, 0.86	0.58, 49%
Generated	*n* = 1,2	96%	67%	93%	0.07, 0.88	0.49, 47%

a Containing one (κ^1^) or two (κ^2^) metal anchors.

b Relative to themselves (that is, not repeating), considering the valid.

c Relative to the training set, considering the valid and unique.

d Average Tanimoto, IntDiv2 coefficients.

e Considering the valid, unique, and novel.

f Average SA score, % with SA score >0.5.

The quality of the generated κ^1^ and κ^2^ ligands was also assessed by considering their diversity (Figure 4), which was analyzed from different angles. The heteroatom distributions were dominated by N, followed by O and P, and the coordination environment distributions were dominated by the N­([C]­[C]) pattern, that is, imine-type ligands like pyridine, followed by the P­([C]­[C]­[C]), that is, phosphines. Regarding molecular sizes, the distributions were close to a broad Gaussian centered nearby a common average value of ∼35 atoms/ligand. In general, the distributions observed for the κ^1^ and κ^2^ ligand sets were similar. The high diversity of the ligands within both sets was also reflected by the small Tanimoto coefficients (Table 1), with average values of 0.08 (κ^1^) and 0.07 (κ^2^). Similarly, the IntDiv2 diversity coefficients were all ∼0.9.^96^ Figure 5 shows a visual perspective on the chemical diversity of the generated ligands; within a random sample of the ten most popular κ^1^ and κ^2^ coordination environments, we found imine, carbene, amine, phosphine, carbonyl, and thiocarbonyl ligands of various sizes and complexities.

**Figure 4:**
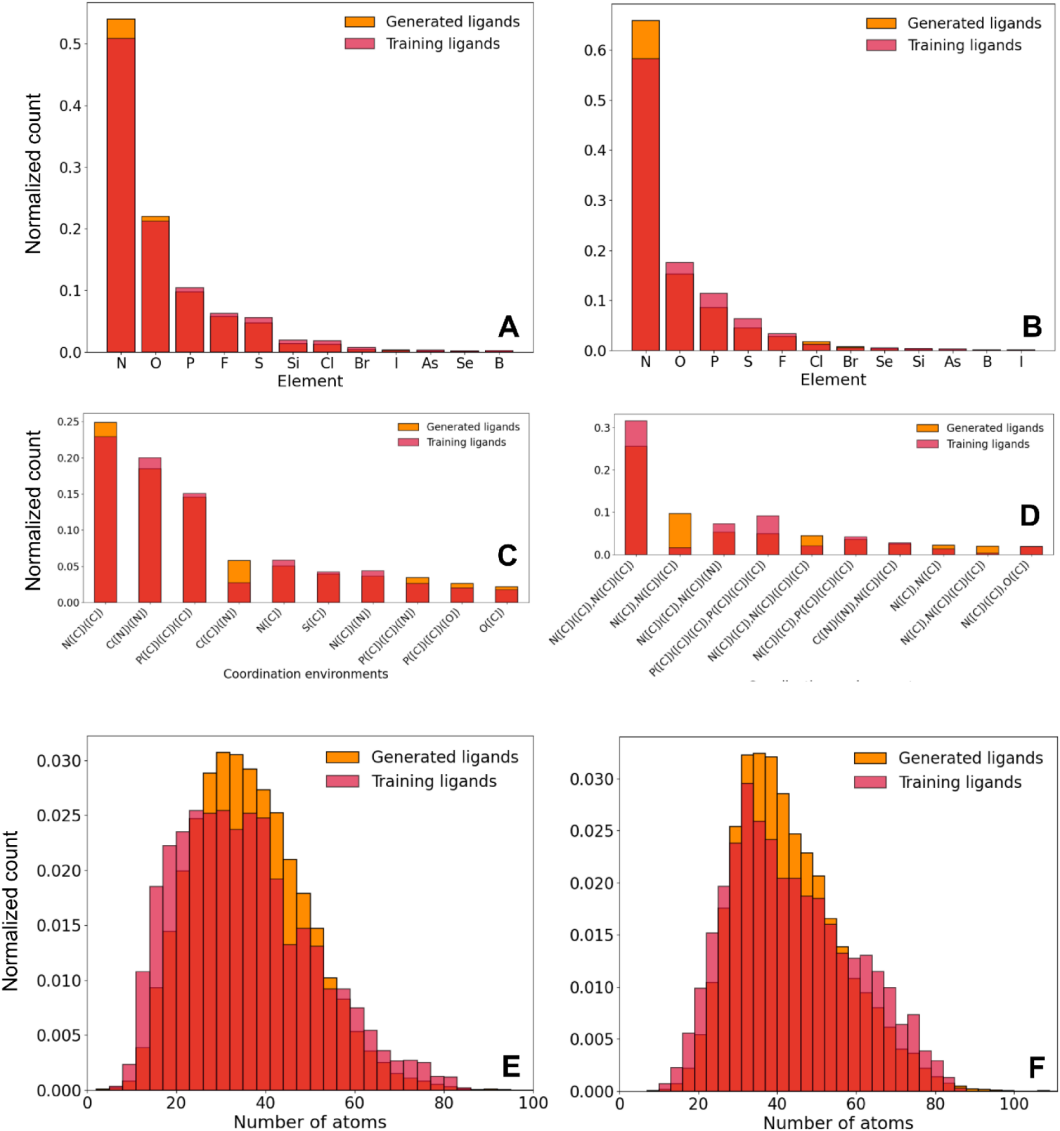
Normalized histograms of the training and unconditionally generated data distributions over the chemical composition (κ^1^ in A, κ^2^ in B), coordination environments (κ^1^ in C, κ^2^ in D), and molecular sizes (κ^1^ in E, κ^2^ in F) of the ligands.

**Figure 5:**
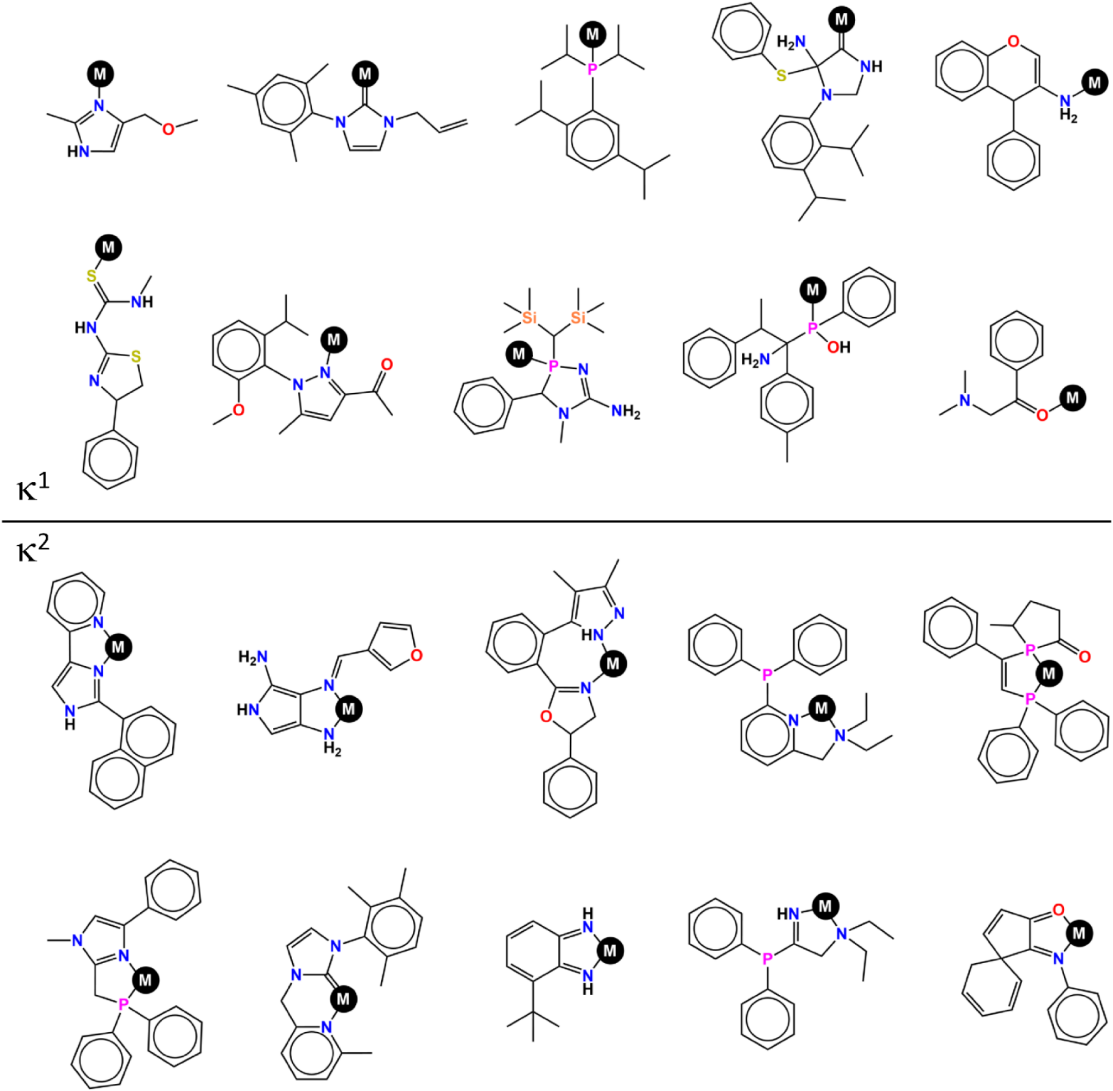
Random selection of unconditionally generated κ^1^ (top) and κ^2^ (bottom) ligands for the ten most common coordination environments, with popularity decreasing from left to right and from top to bottom, following the same order of the distributions C and D in Figure 4.

The experimental feasibility of the generated ligands was assessed with a synthetic accessibility (SA) score. In particular, we used the version derived by Gao and Coley for generative models,^97^ which is based on the original formulation of Ertl and Schuffenhauer.^98^ This score varies from 0 (toughest to synthesize) to 1 (easiest to synthesize). The histogram on the left-hand side of Figure 6 shows the SA score distributions for the training and generated κ^1^ ligands. Both were bimodal, with two tall peaks at the extremes separated by a shallow, wide valley. The peaks at minimal synthesizability, with SA ∈ [∼0.00–0.05], could in principle originate from a significant number of TMCs in the CSD being difficult to make in the lab. However, the intensity of these peaks was also exaggerated by the scarcity of ligand-like molecules in the PubChem database that underlies the calculation of the SA scores,^98^ as shown by the much lower peaks in the distributions of the drug-like subsets (right-hand side histogram in Figure 6). In these subsets, all ligands including some elements that can be regarded as uncommon in organic molecules were excluded. These elements were B, Si, P, As, Se, and I, though others can also be considered (for example, Cl and Br). Similar results were observed in the generation of the κ^2^ ligands (Figures S4 and S5).

**Figure 6:**
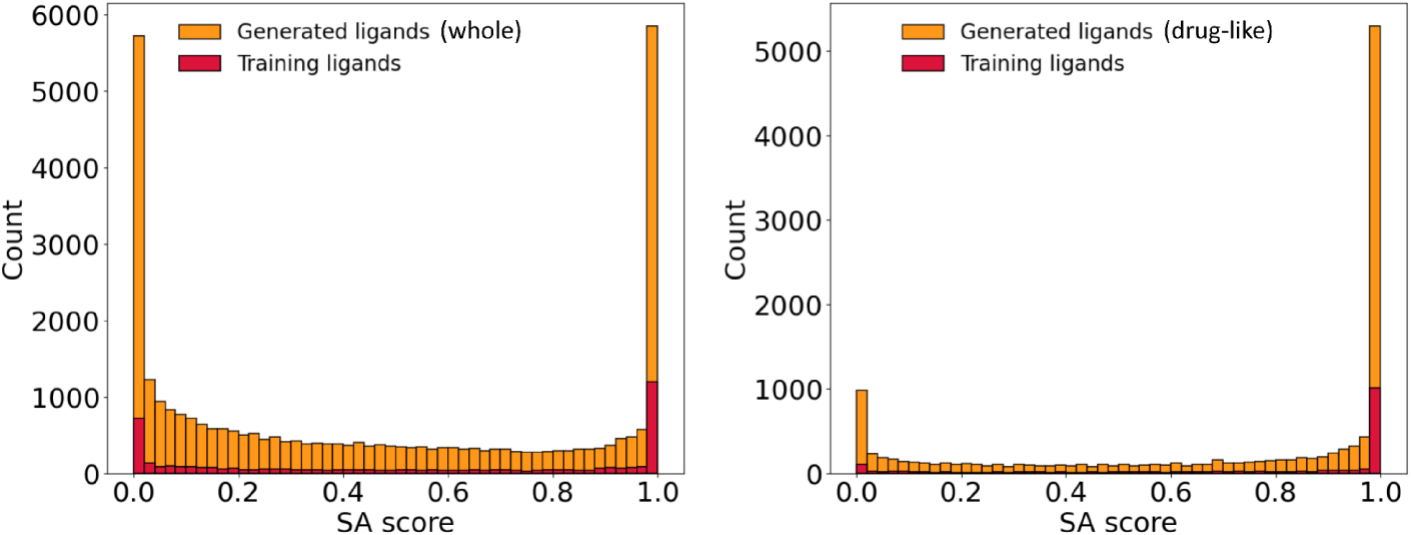
Synthetic accessibility (SA) scores for the whole (left) and drug-like (right) populations of the unconditionally generated κ^1^ ligands.

Using all 9093 ligands available for training, we also optimized a model capable of generating both κ^1^ and κ^2^ ligands. As shown in Table 1, this joint model showed quality metrics similar to those of the separate models, in terms of validity, uniqueness, novelty, diversity, and synthesizability. The property distributions (Figures S6–S8) were similar to those in Figures 4 and 6, with κ^1^:κ^2^ ratios of ∼1:1, in both the training and generated sets.

### Conditional Ligand Generation by TMC Properties

Ligand design is normally guided by target properties that define the whole TMC in which the ligand is installed. For this reason, we developed a JT-VAE model in which ligand generation is conditioned by TMC properties relating to stability and electron density. This first requires labeling the ligands used to train the model with TMC properties.

Using the κ^1^ and κ^2^ ligand sets generated by the unconditional JT-VAEs, we built homoleptic Ir­(I) TMCs based on the square planar [IrL_4_]^+^ and [IrL_2_]^+^ scaffolds, respectively. In particular, for the κ^1^, we used the 15k smallest ligands, whereas for the κ^2^, we used the 10k smallest ligands, in both cases aiming at minimizing steric clashes hindering TMC assembly. The TMC geometries were built automatically with the *molSimplify* program^92^ and, once assembled, they were fully optimized at the DFT PBE-D4/def2SVP level of theory^88–90^ using *ORCA*.^87^ The HOMO–LUMO gap (ϵ) and the iridium atomic charge (*q*_Ir_) of these geometries were computed in single point calculations at the higher PBE0-D4/def2TZVP DFT level.^89–91^ These quantities account for global (stability, ϵ) and local (metal center electron density, *q*_Ir_) properties of the TMCs.

The *molSimplify* success rate in building the geometries was 71% and 65% for the κ^1^ [IrL_4_]^+^ and κ^2^ [IrL_2_]^+^ TMCs, respectively. The deviations from the ideal 100% rate were mostly due to steric clashes enforced by the coplanarity of the four Ir–L bonds. Expectedly, the success rate of the DFT ORCA calculations on the assembled geometries was higher: 93% (κ^1^) and 81% (κ^2^). These success rates and the value of the ligands that failed can be strongly dependent on the nature of the metal center, including the oxidation state and coordination geometry. The computed ϵ and *q*_Ir_ properties appear largely uncorrelated in the scatter plots of Figure 7, in line with their distinct nature. Further, the wide ranges spanned by both ϵ ∈ [1.60, 5.75] eV and *q*_Ir_ ∈ [−0.14, 0.62] *e* (κ^1^), and ϵ ∈ [0.80, 6.20] eV and *q*_Ir_ ∈ [−0.19, 0.65] *e* (κ^2^), show that this set of TMCs spanned an ample region of the property space, consistent with the high diversity of the generated ligands used to build them.

**Figure 7:**
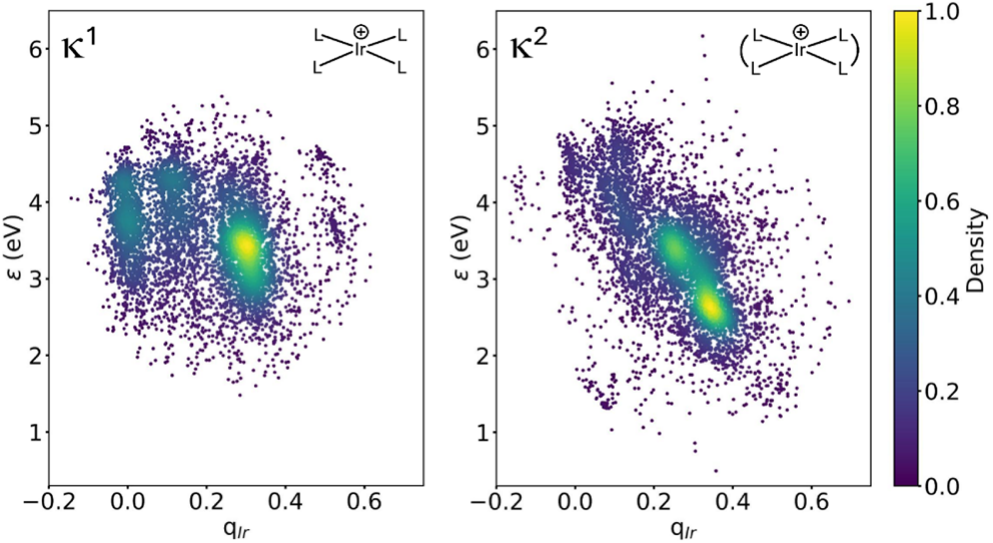
Scatter plots of the *Y* = (ϵ, *q*_Ir_) data for the κ^1^ [IrL_4_]^+^ and κ^2^ [IrL_2_]^+^ TMC sets after excluding 1% and 1.5% of the data points as outliers, respectively, based on an isolation forest model.

The ligands for which the associated homoleptic TMCs passed both the *molSimplify* assembly and *ORCA* optimization steps were compiled and, after excluding the outliers with isolation forests, they yielded two data sets: one including 7068 κ^1^ ligands and one including 4031 κ^2^ ligands, all of them labeled with the *Y* = (ϵ, *q*_Ir_) properties of the corresponding homoleptic TMCs. These data sets were used to train κ^1^ and κ^2^ JT-VAE models that, in addition to reconstruct the input ligands in the generated output, also predict their Y properties with a neural network (Figure 2). Once trained, the models were used for the conditional generation of ligands yielding homoleptic TMCs with optimal *Y* values. In this task, the models encode the SMILES of a metal ligand prompt into the latent space and, by following the gradient, they find a latent vector that maximizes or minimizes one or both Y properties, as requested by the user. Ligand optimization was moderated by Tanimoto coefficient thresholds of 0.20 (κ^1^) and 0.15 (κ^2^), determined in the hyperparameter optimization of the model to enforce a minimum similarity to the initial prompt.

Conditional generation was assessed by considering these eight compass directions in the space of the optimized properties (Table 2): four directions oriented along the orthogonal ϵ and *q*_Ir_ axes (D1–4), and four directions corresponding to the intersections (D5–8), all of them crossing at the center point C at which *Y* = (ϵ, *q*_Ir_) = (0, 0). Twenty optimizations were performed along each of these eight directions starting from prompt ligands sampled from these four extreme regions: R1 and R2, where ϵ and *q*_Ir_, respectively, become maximal, and R3 and R4, where ϵ and *q*_Ir_, respectively, become minimal. The same protocol was applied to 80 ligand prompts sampled from the center C. In this way, the directionality of the single- and dual-objective optimizations could be assessed over the entire Y space, including the min­(ϵ), max­(ϵ), min­(*q*_Ir_), max­(*q*_Ir_), min­(ϵ + *q*_Ir_), max­(ϵ + *q*_Ir_), min­(ϵ – *q*_Ir_), and max­(ϵ – *q*_Ir_) tasks. Table 2 provides the quality metrics of these optimizations.

**Table 2:**
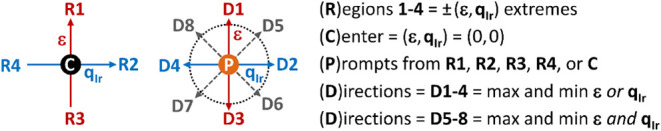
Quality Metrics of the Models Generating Ligands Conditioned to the *Ε* and *q*_Ir_ Properties of the Homoleptic TMCs^a^

Prompt	Similar^b^ →	Valid^c^ →	Unique^d^ →	Novel^e^ →	Verified^f^	Longest D^g^
**κ** ^**1**^ **JT-VAE for homoleptic [IrL**_**4**_ **]**						
R1	42%	100%	90%	92%	49%	D7
R2	66%	100%	75%	89%	63%	D3
R3	73%	100%	87%	89%	55%	D4
R4	57%	100%	92%	92%	68%	D6
C	68%	100%	93%	93%	72%	D3
**κ** ^**2**^ **JT-VAE for homoleptic [IrL**_**2**_ **]** ^**+**^						
R1	47%	99%	97%	97%	58%	D6
R2	57%	87%	97%	91%	51%	D1
R3	66%	83%	95%	96%	62%	D1
R4	59%	89%	88%	95%	49%	D2
C	65%	90%	96%	94%	64%	D2

a All data refers to generated ligands.

b Above TC = 0.20 (κ^1^) or 0.15 (κ^2^) threshold, relative to the initial prompt.

c Containing metal anchor(s); only for those similar.

d Relative to themselves (that is, not repeating); only for those similar and valid.

e Relative to the training set; only for those similar, valid, and unique.

f With normally terminating TMC property calculations; only for those similar, valid, unique, and novel.

g Direction of longest optimization trajectory in *Y* = (ϵ, *q*_Ir_) space. See Supporting Information for further details.

For the κ^1^ model, and regardless of the prompt origin, a significant number of conditional optimizations (42–73%) passed the similarity threshold, yielding 100% validity and high levels of uniqueness (75–93%) and novelty (89–93%) relative to the training set (Table 2). Besides these criteria commonly used in the evaluation of generative models, we also verified that the generated ligands yielded the Y properties that, relative to the prompt, satisfied the min and max conditions defining the eight optimization directions. Verification was done by computing the properties of the homoleptic TMCs at the same DFT level used to label the training data. The verification rates were within the range 49–72%, with the highest rate expectedly associated with the prompts sampled from the center region and, in this particular case, following direction D3 in the longest optimization trajectory.

Similar results were observed with the conditional κ^2^ model (Table 2). Similarity and validity were smaller, likely due to the higher complexity of the bidentate ligands, averaging at 59% and 90%, respectively (61% and 100% for the κ^1^). In contrast, uniqueness and novelty, both averaging at 95%, were larger for the κ^2^ model (87% and 91%, respectively, for the κ^1^), which can be ascribed to another, related factor, namely the lower probability of having repeated ligands when they have two metal anchors instead of a single one. Also in line with the higher complexity of the κ^2^ ligands, the verification % rate was smaller than for the κ^1^, though not by a large margin, with averages of 57% and 61%, respectively. The nature of the longest optimization trajectories was diverse, and, together, the κ^1^ and κ^2^ models produced such trajectories along six of the eight compass directions.

The scatter plot A in Figure 8 includes all conditionally generated ligands in the κ^1^ *Y* space, showing that all four regions plus the center were explored and populated by the JT-VAE optimizer. The other scatters show the longest optimization trajectories in these three different compass directions (Table 2): D3 (B), D5 (C), and D8 (D). In B, all single-objective min­(ϵ) tasks converged into values significantly smaller than those of the prompts. The dual-objective max­(ϵ + *q*_Ir_) task plotted in C was also successful in all cases, though the slope was clearly larger on the ϵ dimension than on the *q*_Ir_. From a Pareto front perspective, the optimization algorithm yielded 12 nondominated points. In contrast, in the max­(ϵ – *q*_Ir_) task (D), optimizations became more erratic. For example, whereas from the R3 and C ligand prompts the optimizations followed the intended direction, from R1 and R2 both properties were minimized; in the former case, the optimization was likely challenged by the condition of maximizing ϵ from a region where this property was already maximal. Similar results were observed for the κ^2^ ligands (Figure S15).

**Figure 8:**
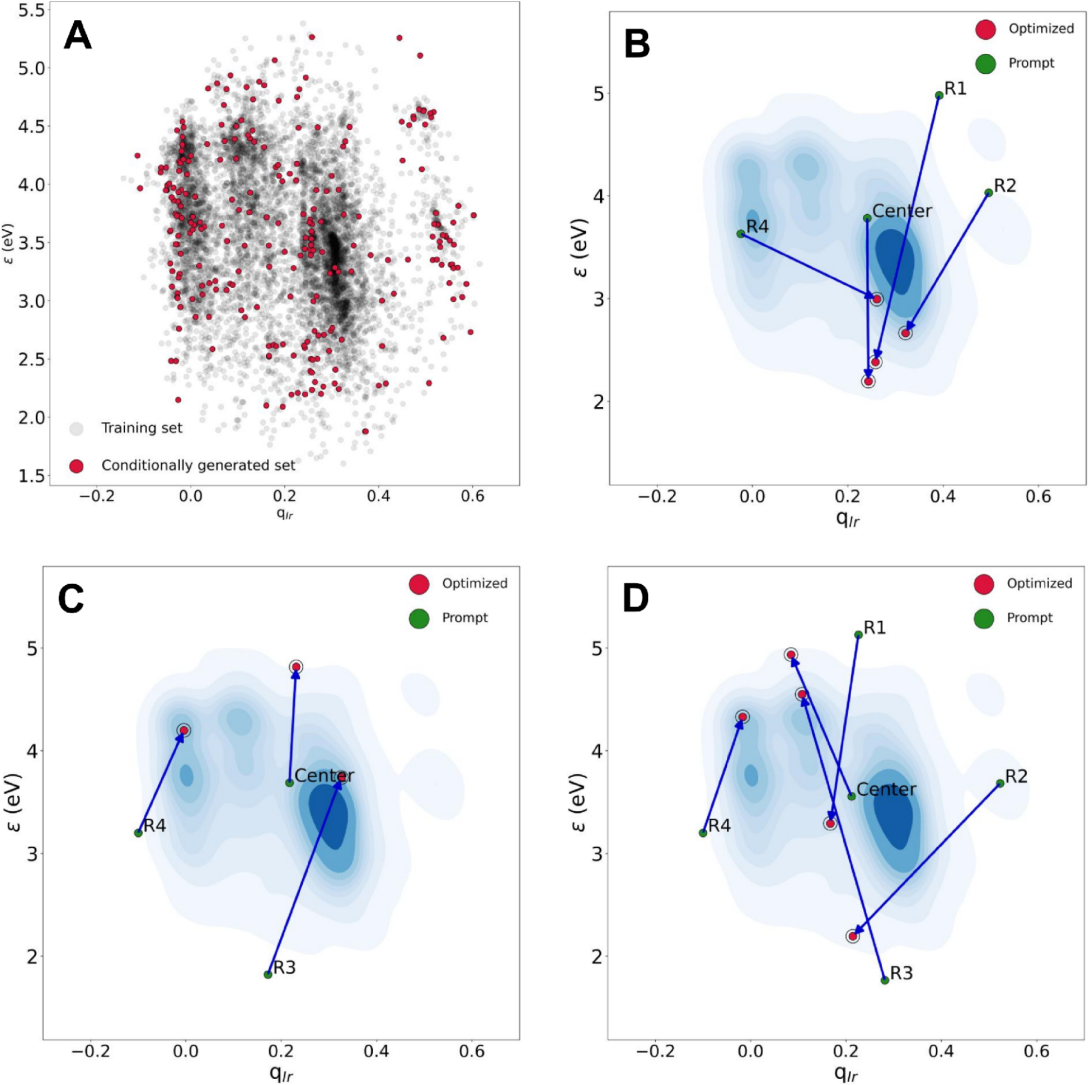
Conditional optimization with the κ^1^ JT-VAE model in *Y* = (ϵ, *q*_Ir_) space: (A) Distribution of all DFT-verified homoleptic TMCs built with conditionally generated ligands; Longest trajectories in the single-objective minimization of ϵ (B), and the dual-objective maximization of both ϵ and *q*_Ir_ (C), and the dual-objective maximization of ϵ and minimization of *q*_Ir_ (D). Contour plots in the background denote training data points density.

Despite being deep generative models, the output of these JT-VAEs can be easily rationalized thanks to the stepwise nature of the gradient descent optimizer. Each step in latent space produces a representation that can be transformed into a ligand using the decoder part of the model. Figure 9 illustrates this concept for the κ^1^ model. For example, when prompted with ligand **L3**, the model transformed this imine into a cyanide ligand fulfilling the conditions requested by the dual-objective max­(ϵ + *q*_Ir_) optimization task, as verified at the DFT level for the corresponding homoleptic TMC. Further, the decoded optimization trajectory showed how the prompt **L3** ligand was transformed over multiple steps, including the replacement of the imine metal anchor by a cyanide and the addition of the electron-withdrawing −CF_3_ group in *ortho* position. Other trajectories, like the optimization of **L4**, were shorter though, in all four examples, the model showed its ability to change the ligand type: from cyanide to amine in **L1**, from imine to carbene in **L2**, from imine to cyanide in **L3**, and from thiocarbonyl to imine in **L4**.

**Figure 9:**
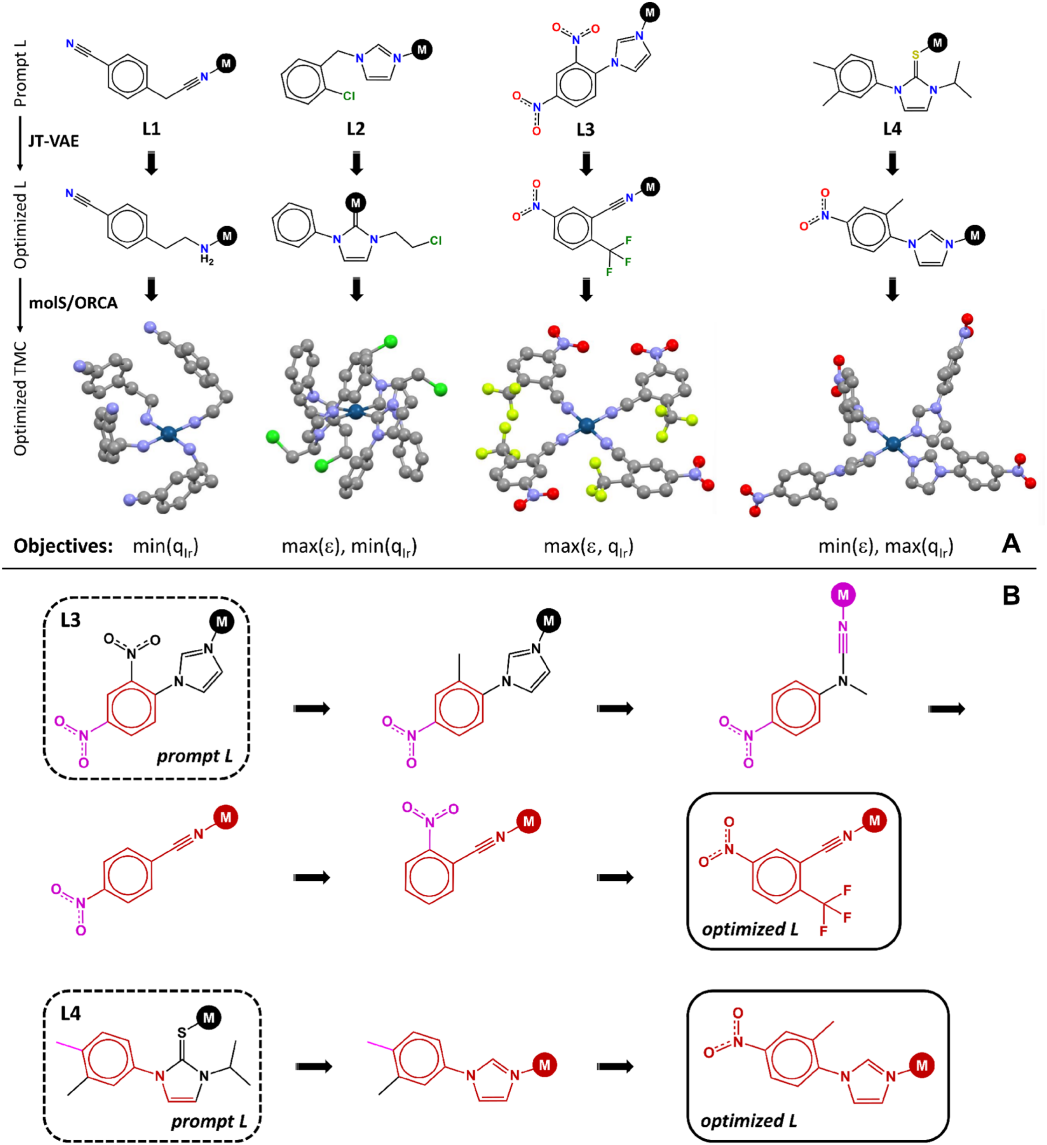
Interpreting conditional optimization with the κ^1^ JT-VAE model: (A) Examples illustrating the optimization of the ligand prompts **L1–4** aimed at four different objectives, and including the DFT-optimized geometry of the corresponding homoleptic TMCs. Element color code: Gray (C), blue (N), dark green (Ir), green (Cl), red (O), light green (F); (B) Examples of long (**L3**) and short (**L4**) optimization trajectories. Color code relative to the optimized ligand: Black (excluded fragments), purple (relocated fragments), red (optimal fragments).

The navigation of the Y space can have an impact on other properties, including the energies and barriers of reactions of chemical interest. For example, the trajectory minimizing ϵ and, to a smaller extent, *q*_Ir_, from region R1 (scatter **B** in Figure 8) transformed the *N*,*N*-heterocyclic core of the ligand (Figure 10) from pyrazole (prompt) to imidazole (optimized), which, in the associated homoleptic [IrL_4_]^+^ TMCs, had a significant impact on the ligand dissociation energy (Δ*G*_LD_) and Ph–Cl oxidative addition barrier (Δ*G* ^‡^_OA_). Both these quantities were reduced in the conditional generation of the ligand by ΔΔ*G*_LD_ = −2.9 kcal/mol and ΔΔ*G* ^‡^_OA_ = −2.2 kcal/mol (Figure 10). These changes can influence the chemistry of the TMC, including its potential use as a catalyst in coupling reactions, since Δ*G*_LD_ and Δ*G* ^‡^_OA_ quantify the ease of opening a vacant site and of activating electrophilic substrates. Besides these properties, ϵ can relate to photochemistry and multireference character, and *q*_Ir_ can relate to electrochemistry and any phenomena depending on the electron density of the metal center. However, the relationships among all these properties, including ligand structure, can be complex due to the diversity of the TMC chemical space.

**Figure 10:**
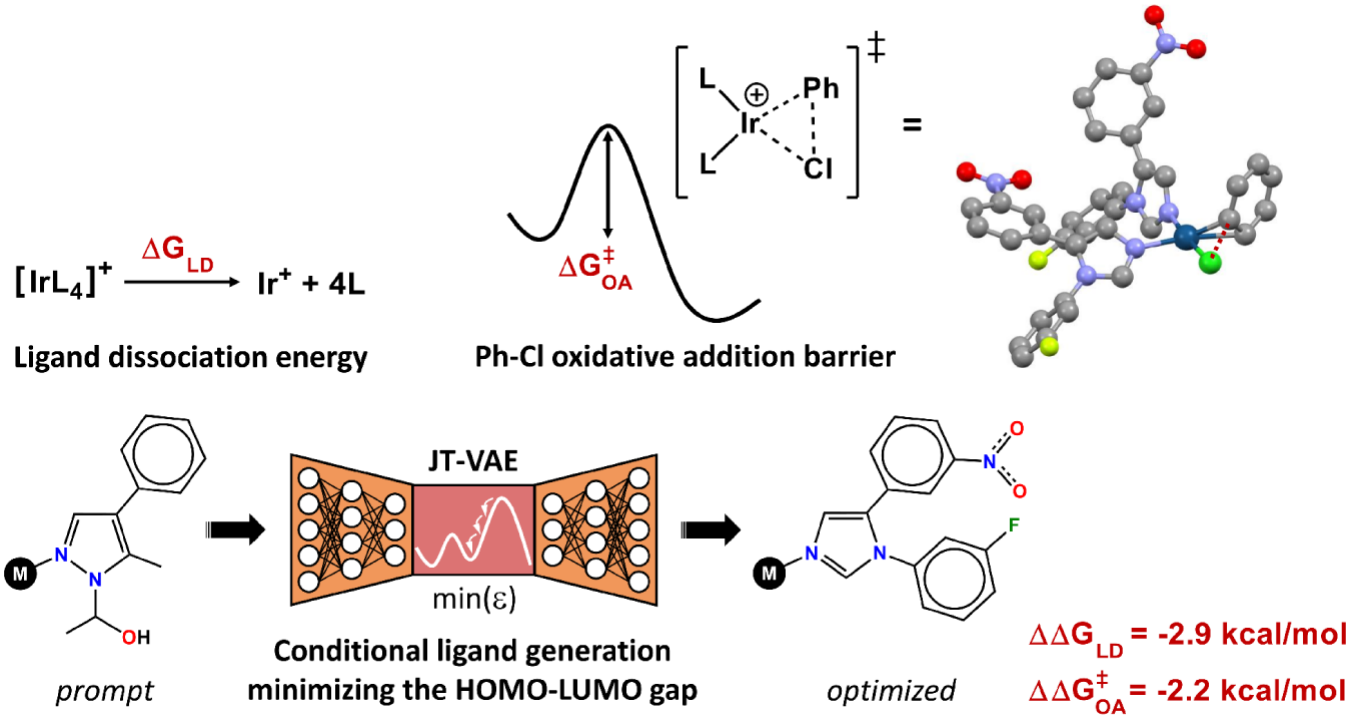
Impact of conditional ligand generation on the dissociation energy (Δ*G*_LD_) and Ph–Cl oxidative addition barrier (Δ*G* ^‡^_OA_) in the associated homoleptic TMC. Element color code: gray (C), blue (N), dark green (Ir), green (Cl), red (O), light green (F).

### Conditional Generation by Free Ligand Properties

We also explored ligand generation conditioned by their intrinsic properties. In particular, both ligand solubility and metal coordination bulkiness are relevant in many TMC applications. For this purpose, the κ^1^ and κ^2^ ligands generated by the unconditional JT-VAE models were labeled with these two properties: (1) log *P*, the octanol–water partition coefficient, computed with *RDKit*,^99^ which quantifies solubility in nonpolar solvents, and (2) Bk_M_, a sum of atomic volumes, which quantifies steric bulkiness around the metal-anchor atom(s). These two properties appear weakly correlated within wide ranges, with log *P* ∈ [−3.60, 7.15], [−3.38, 7.53] and Bk_M_ ∈ [1.50, 24.46], [3.42, 24.66] Å^3^, for the κ^1^ and κ^2^ sets, respectively (Figure 11). Despite the similar maximum values of Bk_M_ in both sets, the κ^2^ has far more instances in the ∼[12–20] Å^3^ range due to the bulkiness adding over two, rather than one, metal anchors.

**Figure 11:**
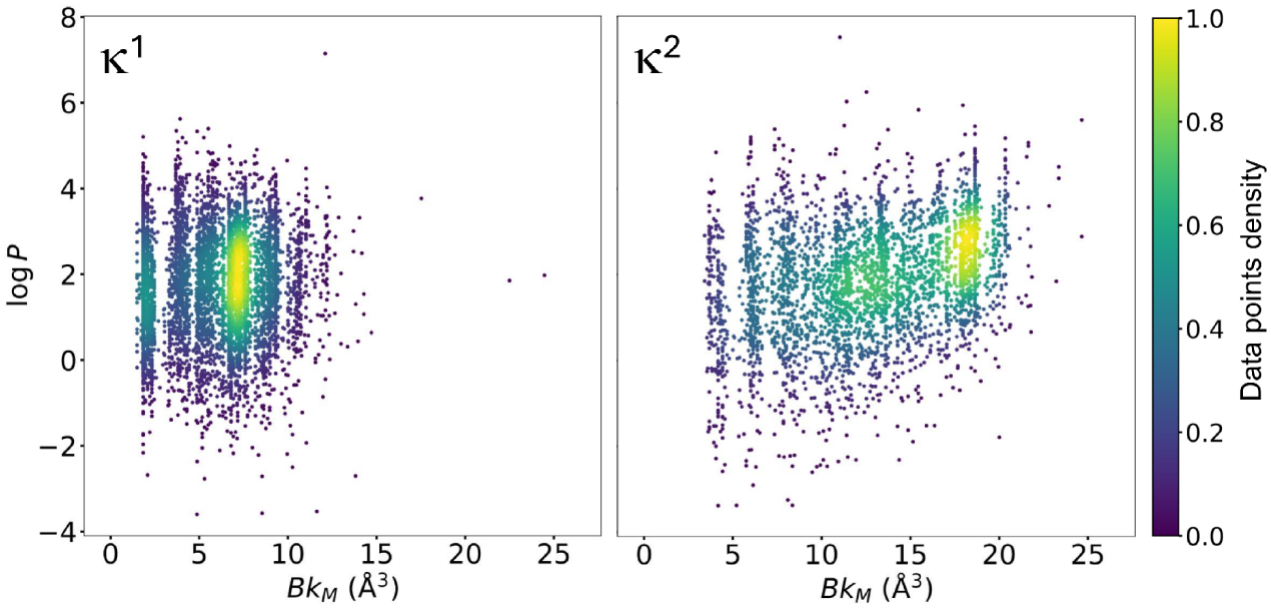
Scatter plots of the ligand coordination bulkiness (Bk_M_) versus the water-octanol partition coefficient (log *P*) for the κ^1^ and κ^2^ ligand sets.

The log *P* and Bk_M_ labels were used to train κ^1^ and κ^2^ JT-VAE generators conditioned by these two properties. Table 3 shows the quality metrics of these models. In general, when compared to TMC-conditioning on ϵ and *q*_Ir_ (Table 2), the performance was better, especially for the verification metric, which is the most demanding. The average verification rates were 78% (κ^1^) and 71% (κ^2^), both significantly larger than those observed for TMC-conditioned generation model (61% and 57%, respectively). This can be attributed to the lower chemical and structural complexity of the free ligands, as well as to simpler relationships between these features and the target properties.

**Table 3:**
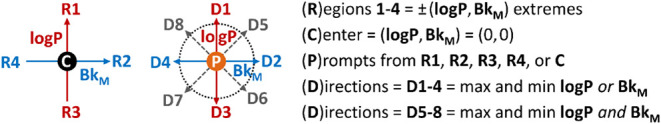
Quality Metrics of the Models Generating Ligands Conditioned by Their Log *P* and Bk_M_ Properties^a^

Prompt	Similar^b^ →	Valid^c^ →	Unique^d^ →	Novel^e^ →	Verified^f^	Longest D^g^
**κ** ^**1**^ **ligands**						
R1	92%	99%	93%	92%	89%	D2
R2	89%	100%	94%	94%	78%	D4
R3	66%	100%	92%	86%	68%	D4
R4	80%	99%	93%	87%	67%	D2
C	83%	100%	88%	90%	88%	D5
**κ** ^**2**^ **ligands**						
R1	92%	99%	89%	92%	77%	D6
R2	96%	100%	90%	90%	76%	D7
R3	56%	100%	73%	91%	64%	D5
R4	78%	99%	88%	88%	65%	D5
C	89%	100%	86%	91%	75%	D5

a All data refers to generated ligands.

b Above TC = 0.20 threshold, relative to the initial prompt.

c Containing metal anchor(s); only for those similar.

d Relative to themselves (that is, not repeating); only for those similar and valid.

e Relative to the training set; only for those similar, valid, and unique.

f With normally terminating free ligand property calculations; only for those similar, valid, unique, and novel.

g Direction of longest optimization trajectory in *Y* = (log *P*, Bk_M_) space. See Supporting Information for further details.

Figure 12 shows four conditional optimization examples, including both κ^1^ (**L6**, **L8**) and κ^2^ (**L5**, **L7**) ligand prompts. These examples show how the ligand property-conditioned JT-VAE models enable inverse design tasks focused on ligand solubility and steric bulk, with input-to-output transformations that can be easily interpreted from a chemical perspective. From **L5**, the min­(log *P*) task reduces the lipophilicity of the prompt ligand by suppressing one of the three phenyl rings, adding also the protic −NH_2_ group. From **L6**, the same objective is met in a similar manner: a fused phenyl ring is entirely replaced by −NH_2_. The **L7** prompt requests the maximization of the ligand bulkiness, which is achieved by fusing a pyridine ring and adding an ortho-tolyl substituent, with both structural changes occurring in the vicinity of the metal anchor. From **L8**, the more complex task of maximizing both log *P* and Bk_M_ is realized by introducing two phenyl rings close to the metal anchor, one of them including an iso-propyl substituent that is added and relocated in the last two steps of the optimization trajectory.

**Figure 12:**
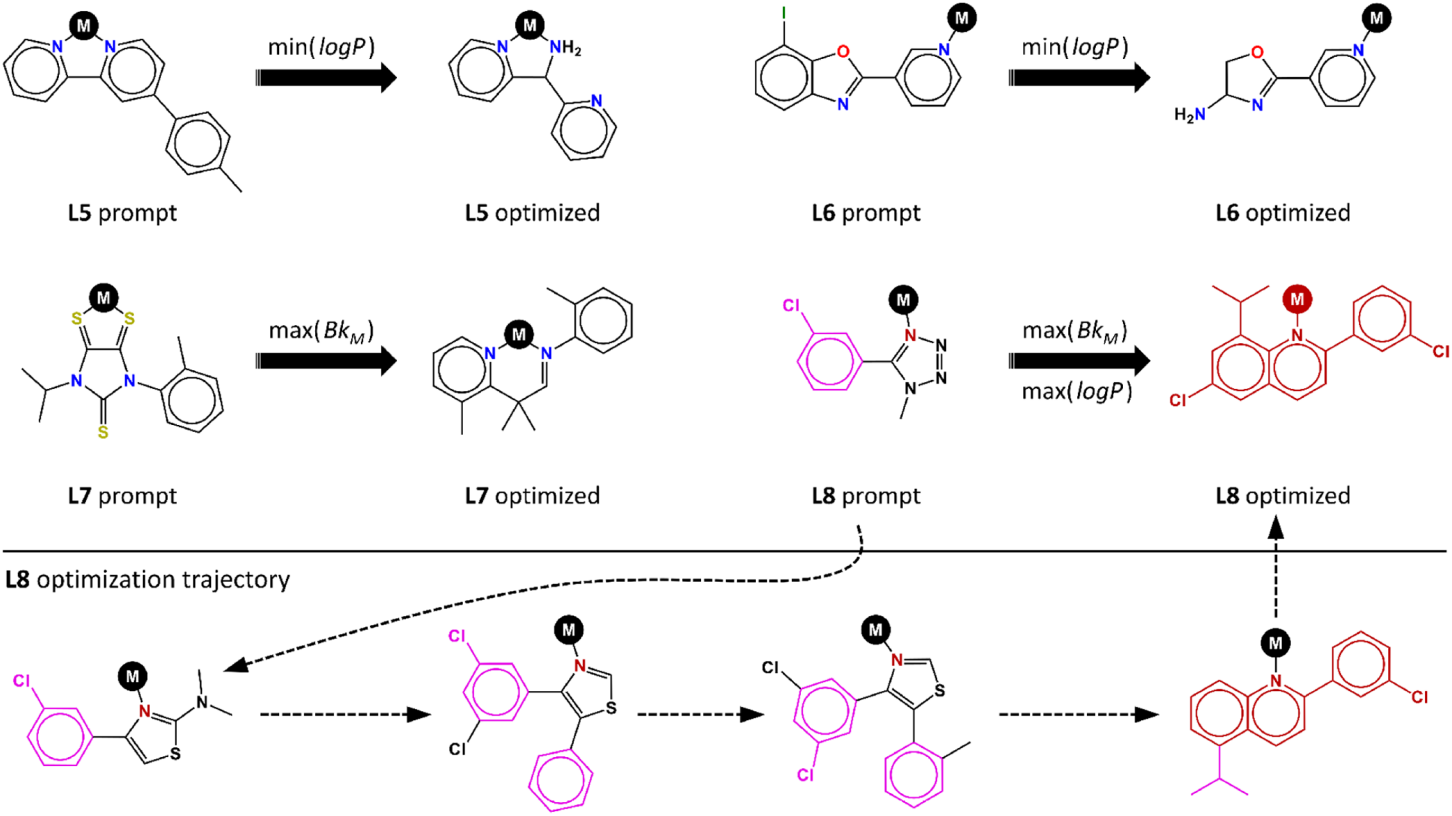
Free ligand generation conditioned by min and max optimizations of the Bk_M_ and log *P* properties. Color code in the **L8** optimization trajectory relative to the optimized ligand: Black (excluded fragments), purple (relocated fragments), red (optimal fragments).

## Conclusions

In this work, we developed a SMILES-based representation for the mono- and polydentate coordination of neutral ligands to transition metals. This representation enabled the encoding and decoding of metal–ligand bonds through generative JT-VAE models. The unconditional models enabled the unbiased generation of κ^1^ and κ^2^ ligands of multiple, diverse types, including imines, phosphines, and carbenes. Whereas validity and novelty were high, both achieving success rates ≥ 93%, uniqueness was moderate, achieving an average value of 65%. With these performance indicators, the models generated 32.8k κ^1^ and 26.3k κ^2^ valid, novel, and unique ligands, augmenting the initial training data by factors of 7.3 (κ^1^) and 5.7 (κ^2^). Besides their size, the generated ligand sets were highly diverse and, in general, they formed distributions over chemical composition, metal coordination, bulkiness, and synthesizability similar to those of the training ligands (Figures 4 and 6), which is valuable given their experimental origin.

The generation of haptic ligands was not explored but is supported by the same metallacycle formulations used for the κ^2^ ligands (Figure 3); for example, a η^2^-(metal–ethylene) bond can be encoded as C1→[Ir]←C1. In principle, this representation is limited to the generation of neutral ligands, though charged ligands could be supported using the [<E ><q>] SMILES notation, where <E> denotes the element symbol and <q> denotes the charge = +, – , ++, etc. However, localizing the ligand charges in the training data and maintaining chemical validity through the JT-VAE latent space might be challenging. Further, the possibility of including more than one metal center in a single representation ([Be] and [Ir]) suggests that, in principle, it should be possible to apply this encoding to the generation of polynuclear TMCs.

It should be noted that our model may not give the most stable coordination isomer of the ligands generated, though, to some extent, this is implicitly encoded and enforced by the experimental origin of the data. For example, the thiocarbonyl κ^1^ ligand in Figure 5 may yield more stable homoleptic TMCs upon coordinating through the amine moieties. This is a complex feature since it depends on the nature of the metal center, including other ligands that can be bound to it, and, for some applications, the TMC isomer of interest may not be the most stable.

The conditional JT-VAEs enabled inverse design tasks aiming at the optimization of two properties pairs: (ϵ, *q*_Ir_) for homoleptic TMCs, and (log *P*, Bk_M_) for the free ligands. In addition to their dual-objective nature, these optimizations were directional, allowing for combining the minimization and maximization of both properties in all possible ways. The intermediate ligands generated along the optimization trajectories could also be valuable if one is interested in nonextreme properties. As expected from the perspective of chemical and structural complexity, the ligand-conditioned tasks were more successful than the TMC-conditioned, with property-verification rates of 78% and 61% (κ^1^), and 71% and 57% (κ^2^), respectively. Higher values might be achieved by either using larger data sets or other representations like SELFIES,^100^ TUCAN,^101^ or the xyz2mol for TMCs.^102^

## Supplementary Material



## Data Availability

Data and code are openly available at https://github.com/uiocompcat/tmcinvdes, including all the training and generated data associated with the JT-VAE models, the code transforming the ligand geometries into metal-anchored SMILES, the code used to curate the training data and optimize and assess the JT-VAE models, and the code used to build, train, and test the JT-VAE models for unconditional and conditional generative tasks.

## Notes

*RDKit* versions, curation of training SMILES, metal–ligand encodings, coordination environments, synthetic accessibility, and the unconditional and conditional models, including DFT computational details, outlier and latent space analyses, optimization trajectories in property space, evaluation metrics, and the definition of the Bk_M_ parameter (PDF)
