# Supplementary material for: A Deep Generative Model for the Inverse Design of Transition Metal Ligands and Complexes
Source: JACS Au. 2025 Apr 23;5(5):2294–308. doi: 10.1021/jacsau.5c00242 (PMC12117439; doi:10.1021/jacsau.5c00242)
Supplement: Supplementary file 1 [file au5c00242_si_001.pdf]

Supporting Information for A Deep Generative Model for the Inverse Design of Transition Metal Ligands and Complexes

Magnus Strandgaard,[†,‡] Trond Linjordet,[†] Hannes Kneiding,[†] Arron L. Burnage,[†] Ainara Nova,[†,§] Jan Halborg Jensen,[‡] David Balcells[†,*]

[†]*Hylleraas Centre for Quantum Molecular Sciences, Department of Chemistry, University of Oslo, P.O. Box 1033, Blindern, 0315 Oslo, Norway;* [‡]*Department of Chemistry, University of Copenhagen, 2100 Copenhagen, Denmark;* [§]*Centre for Materials Science and Nanotechnology, Department of Chemistry, University of Oslo, N-0315 Oslo, Norway;*

E-mail: david.balcells@kjemi.uio.no

Contents

S1 RDKit versions **S3**

S2 Curation of the training SMILES **S3**

S3 Ligand encoding **S5**

S4 Coordination environments **S6**

S5 Synthetic accessibility **S6**

S6 Combined model **S9**

S7 Unconditional ligand generation **S11**

S7.1 JT-VAE model details S11

S7.2 TMC Cartesian coordinates generation from SMILES S13

S7.3 DFT labeling S13

S7.4 Outlier analysis S15

S7.5 Computing labels for free ligands S17

S8 Conditional ligand generation **S18**

S8.1 Latent space analysis S23

S8.2 Free ligand optimization trajectories in property space S24

S8.3 Assessment of conditional ligand generation S24

S8.3.1 Similarity S25

S8.3.2 Validity S25

S8.3.3 Uniqueness S26

S8.3.4 Novelty S26

S8.3.5 Verification and longest trajectory S27

S1 RDKit versions

The JT-VAE implementation is highly dependent on the RDKit version. RDKit 2020.09.1.0 was used in the model environment of this work. Updates to other RDKit versions will require a detailed knowledge of the inner workings of the model. RDKit has received many extensions since 2020; in particular, functionalities related to TMCs. Therefore, we used the 2020.09.1.0 version for the JT-VAE models and the 2023.03.3 version for all other purposes.

S2 Curation of the training SMILES

In order to obtain the SMILES strings of each ligand, a combination of OpenBabel and the rdDetermineBonds module from RDKit was used. The rdDetermineBonds module is based on the xyz2mol package (`https://github.com/jensengroup/xyz2mol`). Depending on the nature a ligand input, OpenBabel and xyz2mol can yield different SMILES. See figure S1 for examples of representation differences in OpenBabel and xyz2mol. A custom procedure that alternates between these two methods based on a set of conditions was designed based on a visual inspection of output molecules from OpenBabel and xyz2mol.

rdDetermineBonds yielded an unusual Zwitterion resonance form of carbenes, where the carbon had a negative charge next to a neighbor atom with positive charge (**1**). In this case, the metal-bound carbon atom of the carbene was determined by using the OpenBabel representation.

Another issue was the fact that OpenBabel was placing radicals on atoms instead of forming double bonds or charged fragments. This is seen in case **2** and **4** in Figure S1. Here xyz2mol was used to assign the charges correctly.

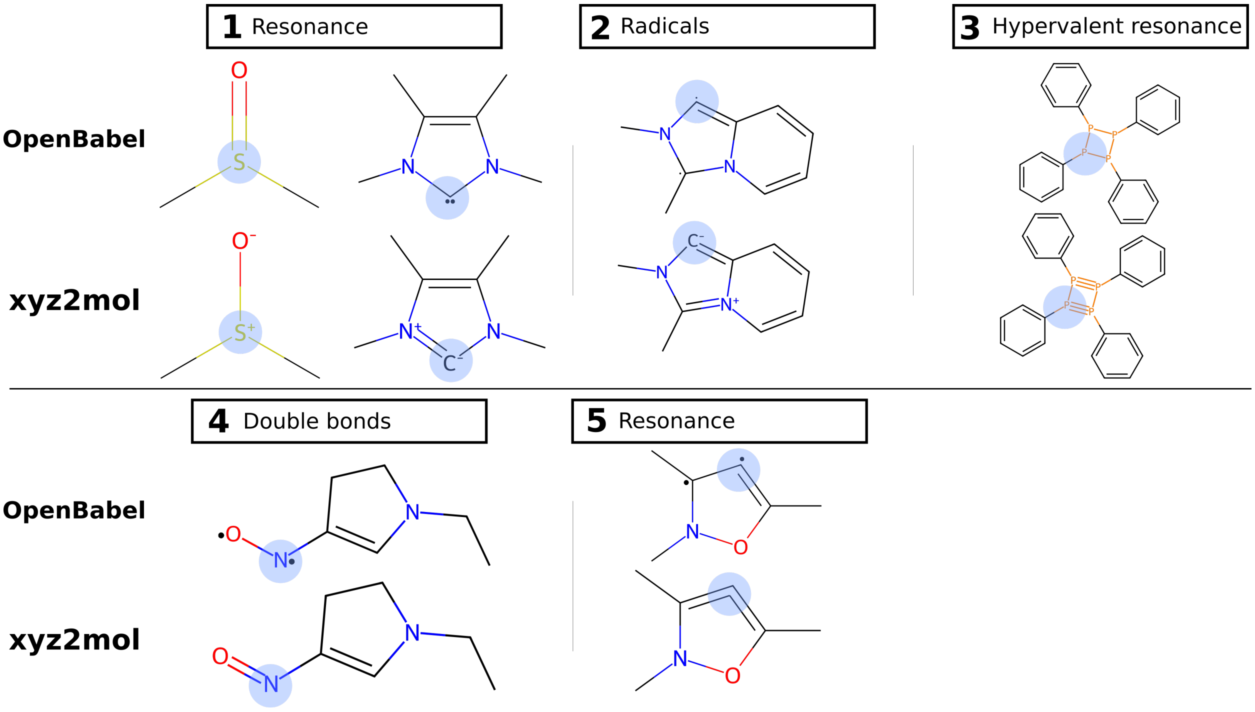

Figure S1: Ligand representation differences between xyz2mol and OpenBabel. All cases are neutral monodentate ligands, where the blue circle marks the metal-coordinating atoms.

Finally, hypervalent compounds like phosphor would also be represented differently. rdDetermineBonds would tend to place high bond orders to P atoms, while OpenBabel would prefer resonance structures with smaller bond orders, as seen in case **3**. In general, the latter were more consistent with the JT-VAE model and thus preferred.

In addition to the representation filters, we also had checks for invalid ligands as seen in the context of TMCs. The tmQMg-L dataset provides the ligand charges and metal-coordinating atoms, which were derived from NBO analysis, with low failure rates of 5% and 3%, respectively.[1] Structures from the CSD can also have errors in the form of missing hydrogen atoms. These errors can lead to incorrect ligand representations which can be hard to detect.

Ligands with coordinating phosphorous atoms with valence above 4 were discarded and ligands with coordinating carbons with valence above 3 were also discarded. This was done to ensure that the quality of the input data to the JT-VAE was as high as possible. This prevented the encoding of ligands that would be invalid when coordinated to a metal. It was

not easy to determine whether an incorrect SMILES representation was obtained based on the xyz2mol approach or if there was an error caused by an incorrect charge assignment in the tmQMg-L library. However, with the filtering of ligands described above we attempted to mitigate such errors.

For example, we observed ligands where the given coordinating atom and charge of a ligand resulted in the coordinating atom being a pentavalent carbon as seen in example **5** in Figure S1. This ligand might be chemically valid in a Zwitterion form and thus originate from an erroneous resonance form. Since no robust method was able to do this type of correction, such ligands were not used for training.

Arsenic and selenium are not supported in xyz2mol and, therefore, OpenBabel was used for all compounds with either of these two elements.

S3 Ligand encoding

After the mol objects and subsequent SMILES were obtained for each ligand, the final step was the metal-coordination encoding of each SMILES string. This was done by attaching substituent atoms to the coordinating atoms. For monodentate ligands, this was done with a lithium atom connected to the coordinating atom through a dative bond. Dative bonds simplified the handling of valence rules since there are not valence constrictions on dative bonds in RDKit.

Carbenes have a large occurrence in the training data and therefore had to be treated carefully. It was found when training the JT-VAE that the metal-bound carbon of the carbene seen as an sp^3 carbon with two hydrogens attached to it instead of a lone pair. Thus, these carbons were encoded as tetra-valent and upon adding Li as the metal anchor they became pentavalent. When generating molecules the JT-VAE would then produce such pentavalent carbon atoms. The encoding was therefore modified to circumvent this issue. Instead of the lithium dative bond encoding, a beryllium atom was connected to the carbene

with a double bond. This ensured that the valence of the carbon atom was filled in the training data and enabled the model to learn carbene environments. This encoding also made it easier to identify carbenes and silylenes in the output generated ligands. After this encoding modification the model was seen to generate beryllium-encoded monodentate ligands which could then be easily treated as carbenes in the post-processing of the output data.

S4 Coordination environments

Due to the encoding of the metal–ligand bonds, we could easily derive the coordination environments of both the κ^1 and κ^2 ligand sets. This was done by expressing the metal-bound atom and its immediate neighbors with SMARTS patterns. Figures S2 and S3 are extended versions of the histograms given in the main text, showing the distributions of the twenty most frequent κ^1 and κ^2 coordination environments.

S5 Synthetic accessibility

Figure S4 and S5 show the SA distributions of two different κ^2 sets: one including all ligands with this coordination mode, and one excluding those containing one or more atoms belonging to this set of elements: `[P,As,Se,Si,B]`. We denote these as drug-like ligands. The main text provides the same Figures for the respective κ^1 sets.

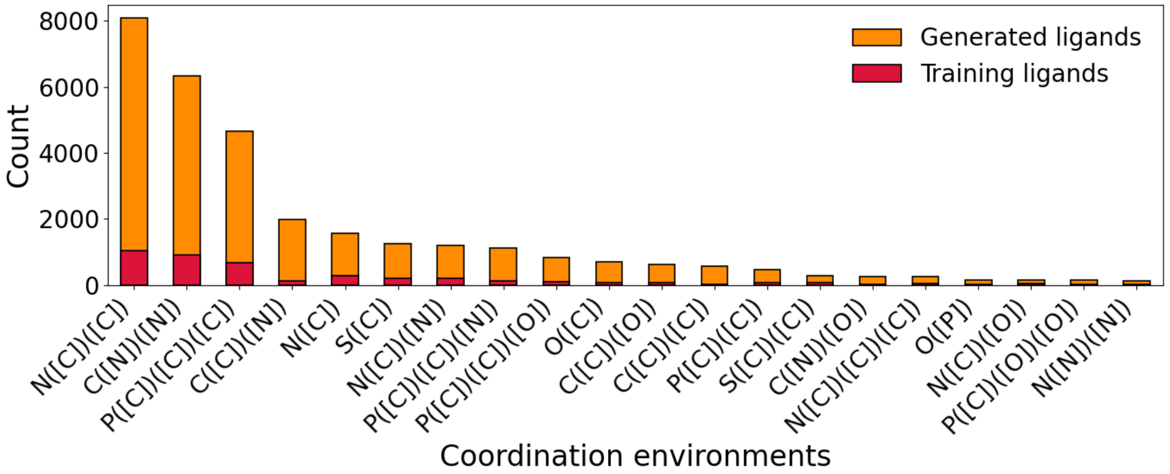

Figure S2: Twenty most popular coordination environments in the generated and training sets of κ^1 ligands. The items on the horizontal axis are SMARTS patterns in which the first atom is the metal-bound.

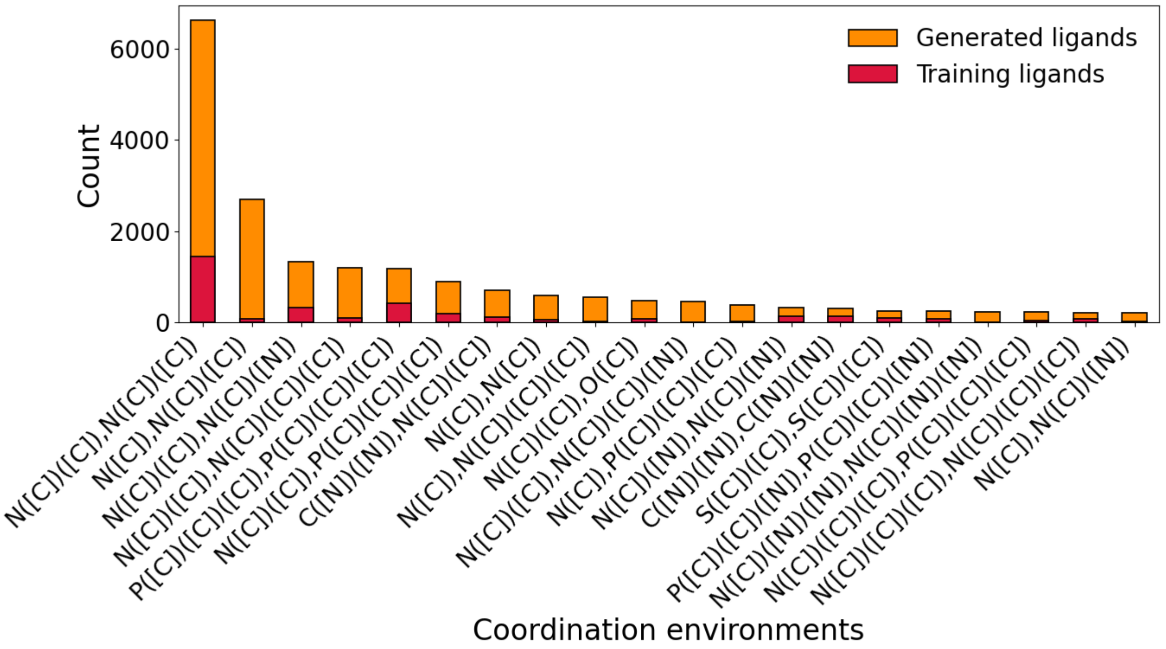

Figure S3: Twenty most popular coordination environments in the generated and training sets of κ^2 ligands. The items on the horizontal axis are SMARTS patterns in which the first atom is the metal-bound.

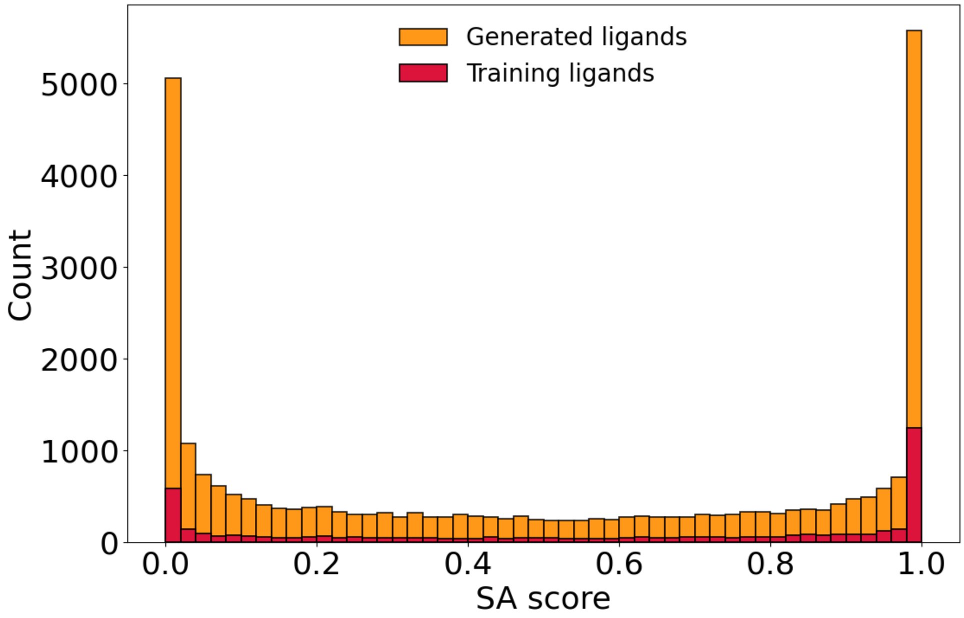

Figure S4: SA scores of the κ^2 ligands.

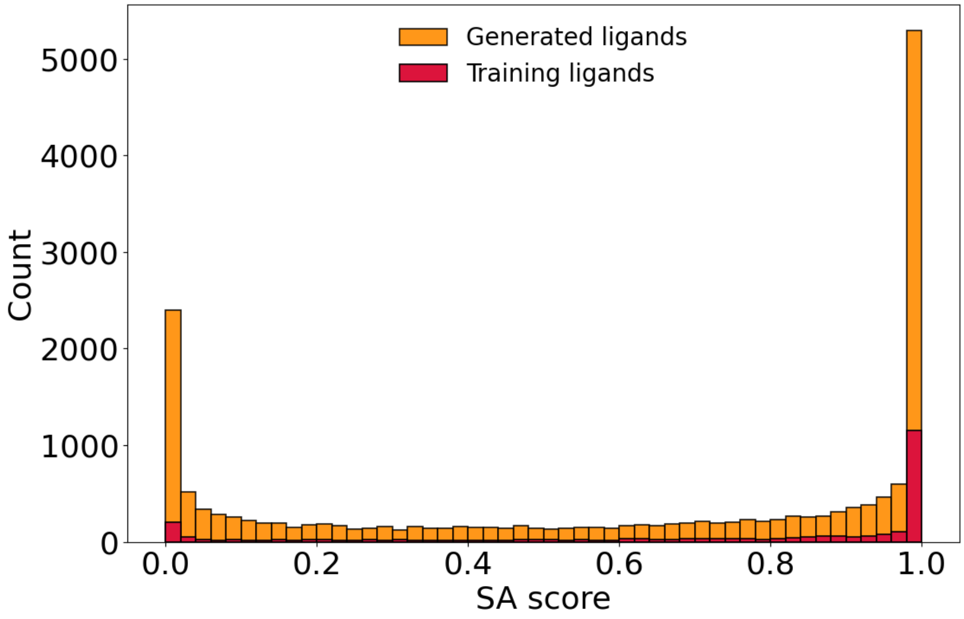

Figure S5: SA scores of the drug-like κ^2 ligands.

S6 Combined model

The following figures show the SA score and chemical distributions of the combined $\kappa^1 + \kappa^2$ model.

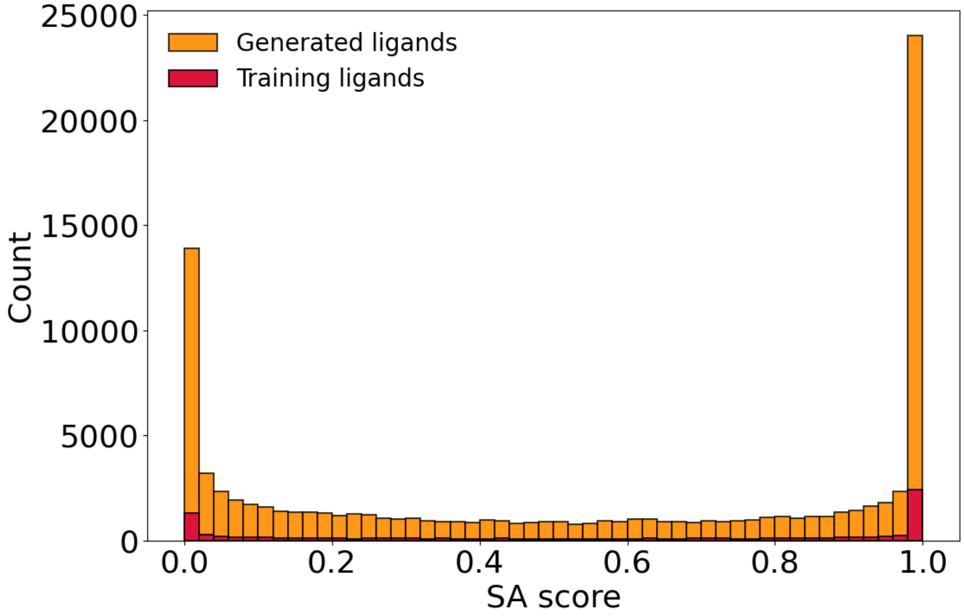

Figure S6: SA scores of the κ^1 and κ^2 ligands.

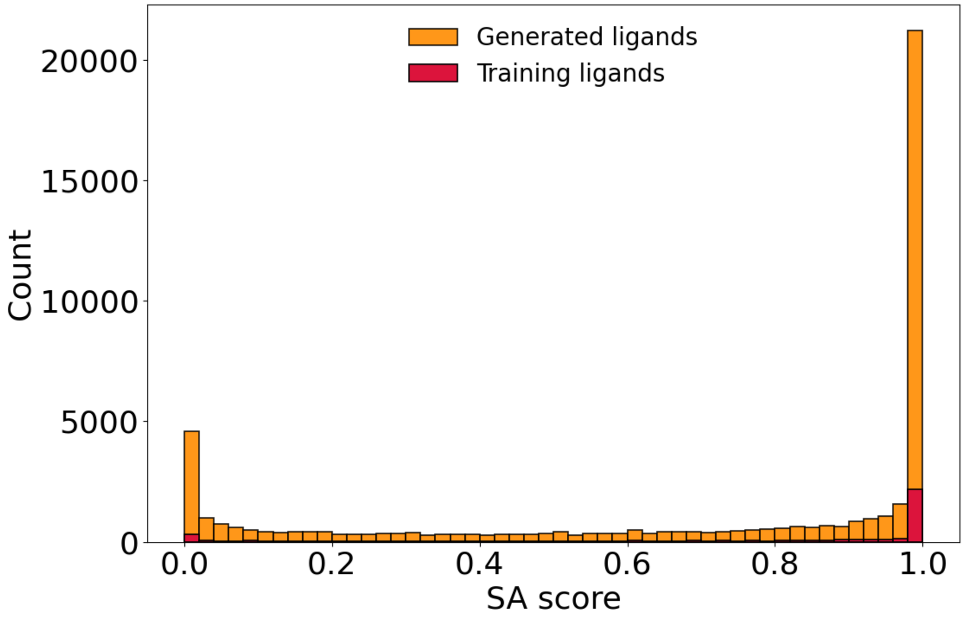

Figure S7: SA scores of the drug-like κ^1 and κ^2 ligands.

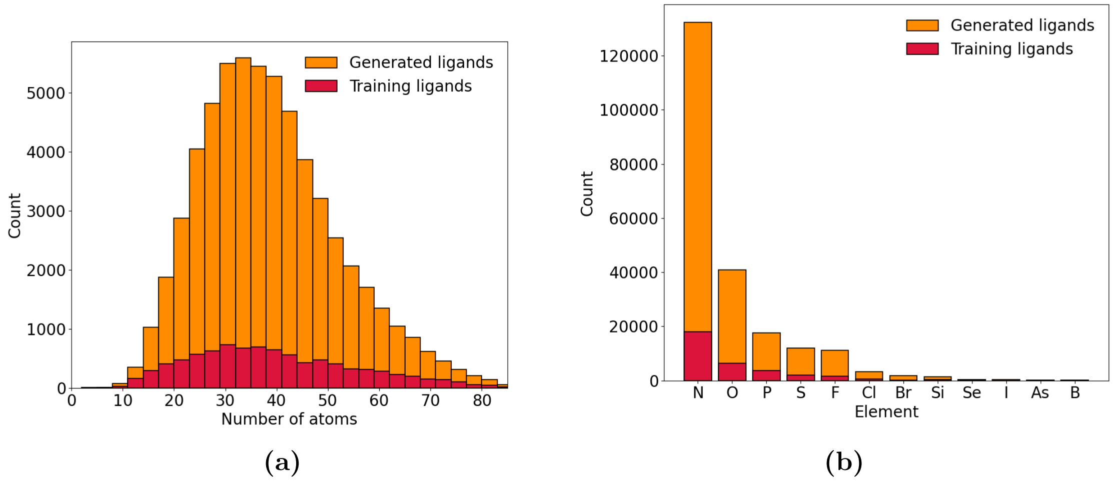

Figure S8: Training and unconditionally generated data distributions over the chemical composition and molecular sizes of the ligands in the combined model.

S7 Unconditional ligand generation

S7.1 JT-VAE model details

For the neutral monodentic κ^1 and bidentic κ^2 JT-VAE models implemented in the study, all available training data was used. In tmQMg-L there were 5210 neutral monodentates. After the curation described in section S2, the final SMILES set had 4511 entries. For the bidentic, these same quantities amounted to 4901 and 4582 ligands, respectively.

The JT-VAE implementation used was based on a fork of the original JT-VAE paper repository,

`github.com/Bibyutatsu/FastJTNNpy3`,

which we forked to create our own model repository,

`github.com/Strandgaard96/JT-VAE-tmcinvdes`,

Here the Python3 translation of the JT-VAE was further modified. The original model architecture and hyperparameters were kept to a large extend except for the following modifications:

- In order to support the encoding of the metal–ligand bonds, the bond feature vector was extended with the `Chem.rdchem.BondType.DATIVE` functionality of RDKit. Further, the three elements used to encode the metal, `[Li,Be,Ir]`, were added to the `ELEM_LIST` list.
- The parameters controlling the annealing phase were changed to suit the size of the training datasets. In particular, warmup = 500, anneal_iter = 1000, kl_anneal_iter = 300, batch_size = 32.

The JT-VAE is inherently guaranteed to produce chemically valid ligands. This raises the question of when to stop model training, as the model will already generate valid molecules from the first iteration. For our purpose, the training process should proceed until the loss converges and the model is able to reproduce the coordination environments of the input data. When models were further trained after loss convergence, they started to produce an increasing amount of ligands already present in the training data (see item list below). The overfitting of the model thus translated into a decrease in the novelty of the output ligands. Another detrimental effect of overfitting was the decrease in uniqueness; that is, there was an increasing number of repeating ligands.

In order to optimize the model used for unconditional ligand generation, we followed this approach: the model was trained for 200 epochs, saving its state at epoch 50, 100, and 200; these three model checkpoints where used to generate three sets of 50,000 ligands for which we determined the percentage of novel items relative to the training datasets and we checked the percentage of generated molecules not found in the training data, finding these results for the κ^1 JT-VAE:

- **Epoch 050:** 96.42% novel ligands.
- **Epoch 100:** 95.48% novel ligands.
- **Epoch 200:** 91.20% novel ligands.

On the basis of these results, we selected the model at epoch 100 as the one having an appropriate balance between loss and novelty. The κ^2 model was also trained for 100 epochs.

S7.2 TMC Cartesian coordinates generation from SMILES

The output of the JT-VAE was SMILES with metal coordination encoded with the `[Li,Be,Ir]` atoms. Thus, a series of steps had to be taken to go from the generated SMILES to 3D Cartesian coordinates of the homoleptic Ir(I) square planar TMCs. The `[Li,Be,Ir]` atoms were first removed using the functionalities of the RDKit. In this procedure, the coordinating atoms were identified as those directly bound to the metal anchors. The ligands were next passed to molSimplify, using the following command to generate the 3D geometries:

```
molsimplify -skipANN True -core Ir -spin 1 -geometry sqp -lig LIGAND_SMILES
-coord 4 -ligocc 4 -smicat COORDINATING_ATOM_IDX -oxstate 1
```

As the input data contained ligands extracted from a wide range of geometries, it was expected that a significant amount of ligands would fail to pass this procedure due to steric clashes of bulky ligands in the homoleptic square planar coordination geometry. In this manner, the "molSimplify pass" already acted as a first screening filter. The ligands for which molSimplify succeeded to generate the TMC geometry were further considered for the DFT labeling.

S7.3 DFT labeling

DFT calculations were performed with ORCA5, using the D4 dispersion correction. The PBE/def2SVP level was used for structure optimization, followed by singlepoint calculations at the PBE0/def2TZVP level, yielding the $Y = (\epsilon, q_{Ir})$ labels. Structures were fully optimized without any geometry or symmetry constraints. The TMC datapoints are shown in Y space in the scatter plot of Figure S9 for both the κ^1 $[IrL_4]^+$ and κ^2 $[IrL_2]^+$ sets, including outliers. Figure S10 shows only the $[IrL_4]^+$ set, without outliers, and indicating the ten most frequent coordination environments.

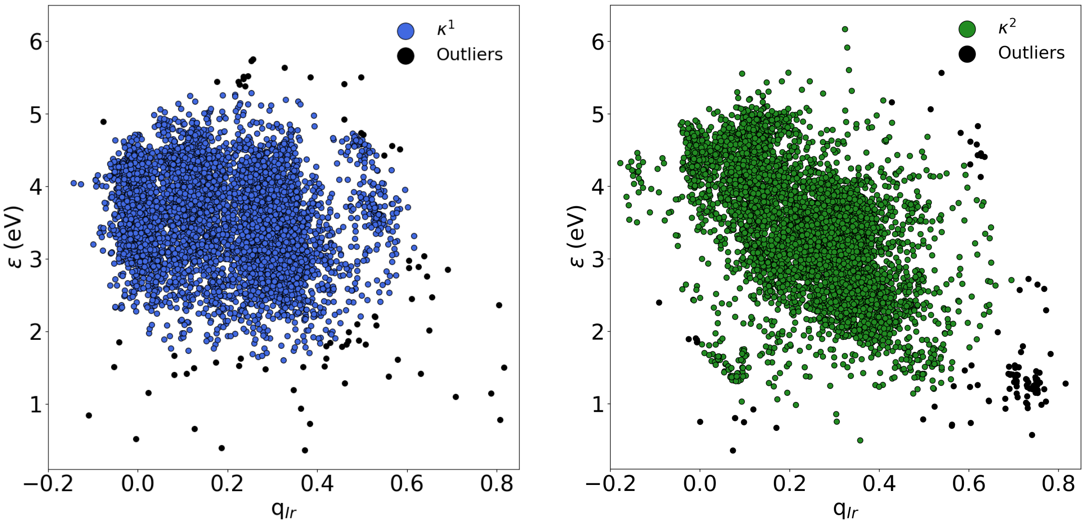

(a) **(b)**

Figure S9: Scatter plot of DFT-labeled κ^1 and κ^2 ligands, with outliers marked in black.

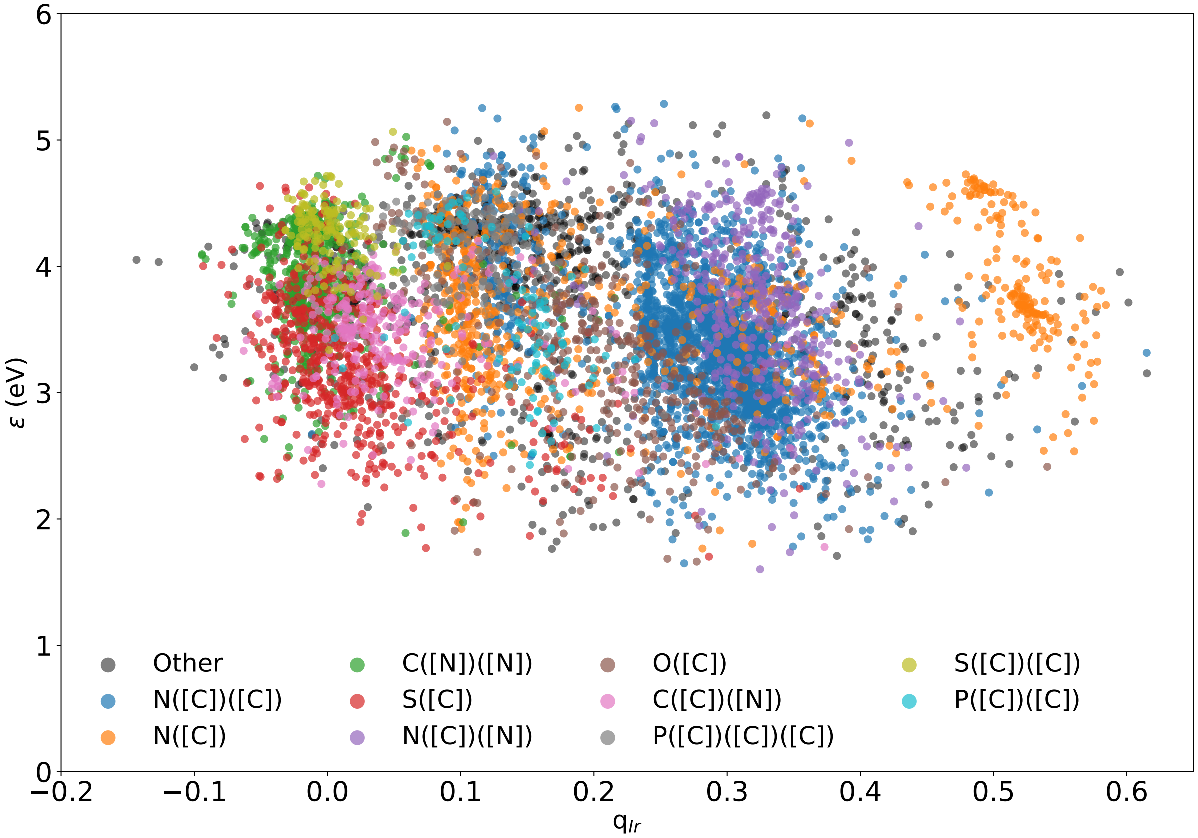

Figure S10: Scatter plot of the κ^1 $[IrL_4]^+$ set with outliers removed and with colors indicating the top 10 highest occuring coordination environments.

S7.4 Outlier analysis

In the Y = (ϵ, q_{Ir}) distributions, 1.0% (κ^1) and 1.5% (κ^2) of the datapoints were excluded as outliers using an isolation forest model and they are thus not shown in Figure 7. The full distributions are shown in Figure S9. The visual inspection of the outliers revealed the presence of several TMCs with geometric distortions, including the increase of the denticity κ^n order ($n = 1 \rightarrow 2$ and $n = 2 \rightarrow 3$), ligand rearrangements (decoordination, fusion, and fragmentation), and changes in the coordination number (from 4 to 5 or 6) and geometry (from square planar, to tetrahedral, square pyramidal, or octahedral). Figure S11 provides examples of these distortions, which, in general, were caused by ligand$\cdots$ligand steric clashes and were thus more common in the κ^1 $[IrL_4]^+$ set (63% of the outliers) than in the κ^2 $[IrL_2]^+$ (14% of the outliers).

We explored with more detail the nature of the TMCs among the 1.0% ($[IrL_4]^+$) and 1.5% ($[IrL_2]^+$) outliers found by the isolation forests in the (q_{Ir}, ϵ) space built with the unconditionally generated κ^1 and κ^2 ligands. The DFT-optimized geometries of all these outliers were inspected visually. Figure S11 provides four examples for each of the two TMC sets. In the $[IrL_4]^+$ set, we observed different types of deviations from the square planar coordination geometry. For example, in the top row of Figure S11, left to right: $\kappa^1 \rightarrow \kappa^2$ isomerization, causing either the decoordination of other ligands or an increase of the coordination number, ligand fusion by bond rearrangement, and tetrahedral iridium centers. In the $[IrL_2]^+$ set, we observed similar and distinct geometric deviations. For example, in the bottom row of Figure S11, left to right: $\kappa^2 \rightarrow \kappa^3$ isomerization of either one or both (octahedral iridium) ligands, $\kappa^2 \rightarrow (\kappa^1, \eta^2)$ isomerization, and ligand fragmentation.

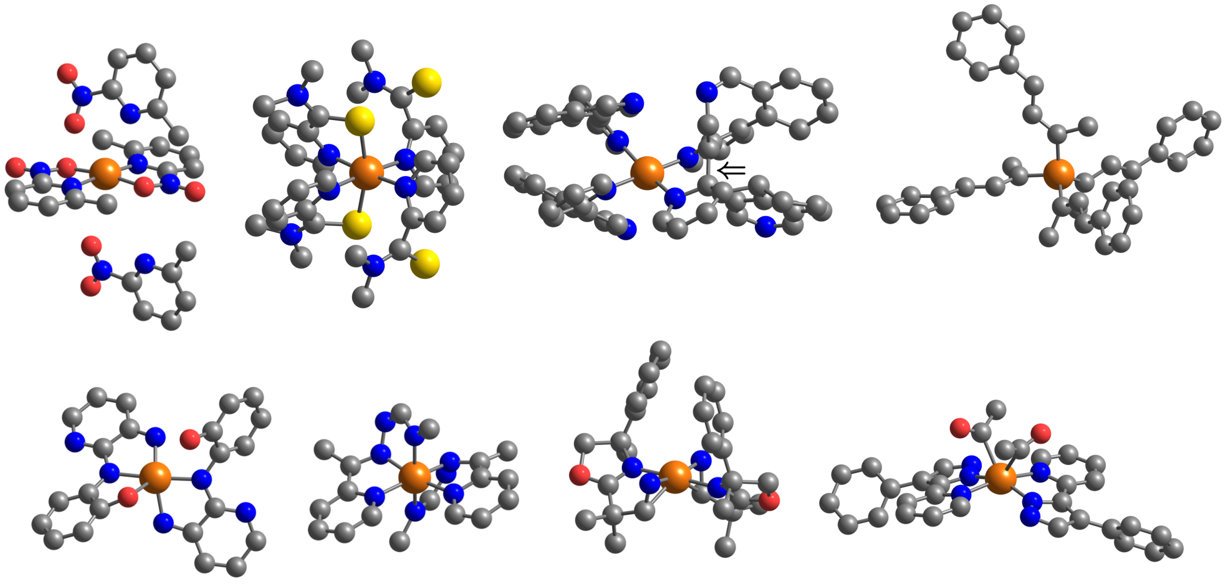

Figure S11: $[IrL_4]^+$ and $[IrL_2]^+$ TMC outliers in the (q_{Ir},ϵ) space built with the unconditionally generated κ^1 (top) and κ^2 (bottom) ligands, respectively. The arrow marks a chemical bond fusing two ligands. Hydrogen atoms were removed for clarity. Element color code: Orange (Ir), blue (N), red (O), grey (C), yellow (S).

Most of the geometric deviations observed in the $[IrL_4]^+$ and $[IrL_2]^+$ outliers appear to be caused by steric clashes in the initial geometries passed to *ORCA* for their DFT geometry optimization. In line with this, the proportion of geometrically distorted TMCs was significantly larger in the $[IrL_4]^+$ set (63%) than in the $[IrL_2]^+$ set (14%), due to the smaller number of $L \cdots L$ contacts. Further, in the $[IrL_2]^+$ set, the chelating nature of the κ^2 ligands, which are often also more rigid, due to, for example, fused rings, hampers certain distortions, including ligand dissociation.

Besides these geometric distortions, we detected ligands with missing hydrogen atoms. For example, sulphur-bound ligands that could only be closed-shell and neutral after adding a hydrogen atom. This issue, which was observed in only 11% of the $[IrL_4]^+$ and $[IrL_2]^+$ outliers, seemed to originate from the interpretation of the ligand SMILES strings passed to *molSimplify*. Due to even n in $[IrL_n]^+$ ($n = 2, 4$), these ligands yielded TMCs consistent with a closed-shell cationic state, which, in some cases, produced stable geometries. However, we also observed ligands with missing hydrogens that broke into fragments during the DFT optimization of the associated TMC.

S7.5 Computing labels for free ligands

While DFT labeling as described above reflected the properties of homoleptic TMCs as determined by a single ligand, it is also interesting to consider the properties of the free ligand itself. In particular, the solubility and steric bulkiness of the free ligand are of interest. Solubility was quantified with the water-octanol partition coefficient, $logP$, by using the corresponding *RDkit* functions on the free ligand, after mapping its SMILES encoding into a `mol` object.

Given the extreme diversity of the ligands used for training and thereafter generated, we defined a ligand bulkiness descriptor, Bk_M, that does not depend on the nature of the metal or other surrounding ligands, which yet refers to the ligand atoms acting as metal anchors. For any given ligand, we take the encoded SMILES representation, map it into a `mol` object with *RDkit*, adding all hydrogen atoms and embedding it into the 3D Cartesian space, from which an `xyz` block is created. The `mol` is also decoded to remove the metals but tracking their indices as well as those of the metal anchor atoms, so that the former can be removed from the `xyz` and the latter found in it. For each metal anchor atom a_{ma}, we take all nearby non-coordinating atoms $a_{nc} \notin \{a_{ma}\}$ within a sphere of radius equal to 2.5 Å. We then sum all the covalent volumes of a_{nc}, computed as that of the sphere defined by the respective element covalent radius, $r_{a_{nc}}$, as recorded at `https://github.com/hkneiding/atom-property-table/`. Following these definitions, Bk_M is computed for any ligand with this equation:

$$Bk_M = \sum_{a_{ma}} \sum_{a_{nc}} \frac{4}{3} \pi r_{a_{nc}}^3 \tag{S1}$$

S8 Conditional ligand generation

For conditional generation, we used the same approach of Jin and co-workers.[2] An additional neural network was added to the JT-VAE architecture to predict Y from latent space. During training, the MSE of this property predictor was added to the loss. We found that the JT-VAE codebase was modified after the publication of the original model, which made the conditional generation not functional in the latest version of the code. Our repository contains the adjusted JT-VAE code that makes the property prediction work with the modified version of the codebase. Additionally, the original code only supported the optimization of a single property. We extended the code for dual-objective optimization. In principle, this can be trivially extended to higher dimensions, adding additional target properties. The conditional JT-VAE was trained with the same hyperparameters used for the unconditional model.

The trained conditional JT-VAE model allowed us to perform directional sampling in latent space. A prompt ligand is first encoded and we then obtain the gradient of the predicted properties with respect to the associated latent vector. Next, we perform gradient descent with step size α_i according to equations (S2) and (S3). α is the step size along the gradient. We normalized both gradients to be able to consistently control the distance traveled along the optimization trajectory for each property. α is set to a constant value obeying this condition: $\alpha_2 = 1.5 \cdot \alpha_1$, with $\alpha_1 = 1.5$. When optimizing in both directions, it was observed that the charge gradient was dominating, resulting in only the charge being optimized, which is the reason behind the ratio between the two α parameters. When performing the conditional optimizations discussed in the main manuscript, we took 250 steps along the gradient using these α_1 and α_2 step sizes.

$$z^*_{q_{Ir}} = z_{prompt} \pm \alpha_1 \cdot \frac{\delta q_{Ir,pred}}{\delta z} \tag{S2}$$

$$z^*_{\epsilon} = z_{prompt} \pm \alpha_2 \cdot \frac{\delta \epsilon_{pred}}{\delta z} \tag{S3}$$

When we applied equations S2 and S3, we obtained latent vectors that according to our model should represent ligands that, when converted to $[IrL_4]^+$ TMCs, have optimal properties in the two dimensional Y property space. We feed the new latent vectors through the decoder to obtain the optimized SMILES. As an example, we take a labeled point in the training data that has a small ϵ value and sample the latent space, according to equation (S3), for latent vectors that increase this property. A visual illustration of this procedure is shown for an example carbene ligand in Figure S12.

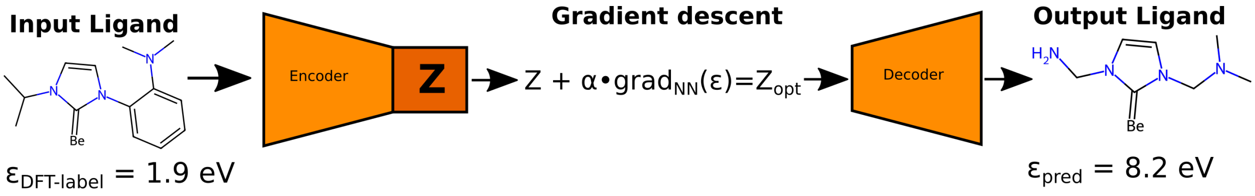

Figure S12: Illustration of how the trained conditional JT-VAE is used to optimize ligands. In this case, we attempt to maximize ϵ.

There are multiple ways of sampling along the gradient. The approach we use is a modified version of the original implementation.[2] It is based on sampling multiple latent vectors along the gradient, with an iterative algorithm selecting which vector to use. Essentially, this algorithm finds the sampled latent vector that is furthest along the gradient relative to the starting point and yet satisfies the similarity condition set by the Tanimoto coefficient cutoff of 0.2 for monodentates of 0.15 for bidentates.

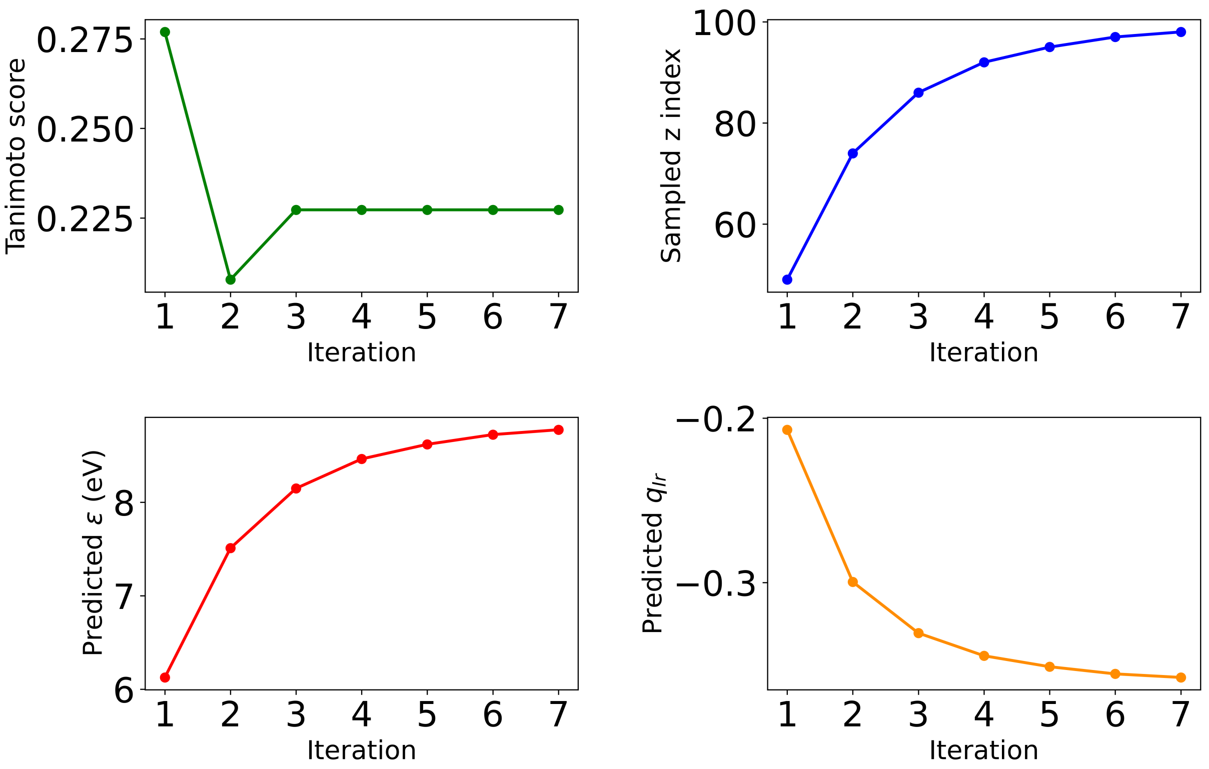

Figure S13: Illustration of the iterative algorithm used to sample from latent space of the conditional JT-VAE. The x-axis indicates each step in the algorithm. The blue curve indicates the step in latent space. This is the sampling associated to the conditional generation task shown in Figure S12.

Figure S13 shows the latent space sampling done to obtain the optimized carbene ligand of Figure S12. Here we sampled 100 latent vectors by starting from the representation of the input ligand and adding the normalized negative gradient times the step size to the gradient 100 times (Equation (S3)). The gradient is recomputed after each step. In the first iteration of the algorithm, we go to the middle of the sampled space which corresponds to the 50th sampled z vector (iteration 1 on the blue line). We want to evaluate the model's ability to modify an existing structure, and not just replace the whole ligand with a completely new one, which would be a much easier task as the model would then only need to sample from the extremes of the learned distributions. This is controlled by the Tanimoto coefficient cutoff. If a sampled latent vector results in a molecule that has a lower similarity to the

original ligand, it is rejected since this indicates that we have likely moved too far along the gradient and need to inspect other latent vectors closer to the starting point.

In the case shown in Figure S12, we are above the Tanimoto cutoff and continue to higher indices of the sampled vectors, which means further from the starting point. This is also illustrated by the red line where the ϵ prediction is observed to increase. We can see how the Tanimoto score of the predicted molecule from iterations 3-6 is unchanged, even though the property predictions are significantly different. This is one reason for not checking every latent vector along the gradient, as changing the latent vector slightly by a gradient step gives a new property prediction but the molecule corresponding to this prediction is often unaltered. This is likely a reflection of the limited training data causing neighboring z vectors to map into the same SMILES strings.

The starting prompt ligands used for the monodentate conditional generation are shown on top of the black training points in Figure S14.

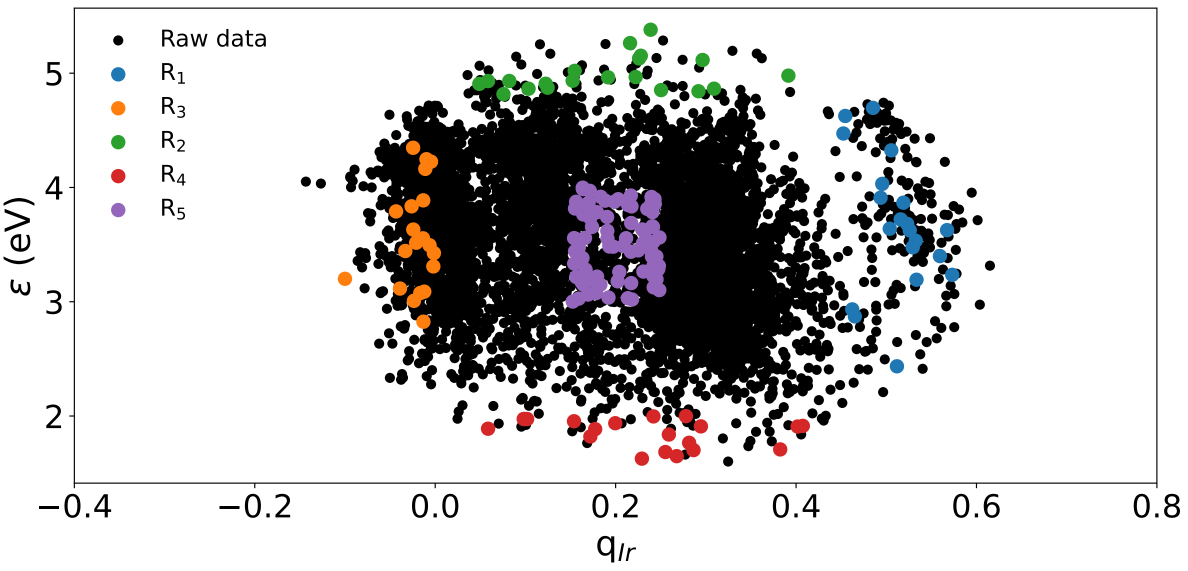

Figure S14: Illustration of the 160 monodentate starting samples for directional optimization in latent space. Black points indicate the training data used for the conditional monodentate model.

Figure S15 shows four different examples of TMC-conditioned ligand optimization in the $Y = (\epsilon, q_{Ir})$ property space, including both single- and dual-objective tasks along the longest trajectories converged.

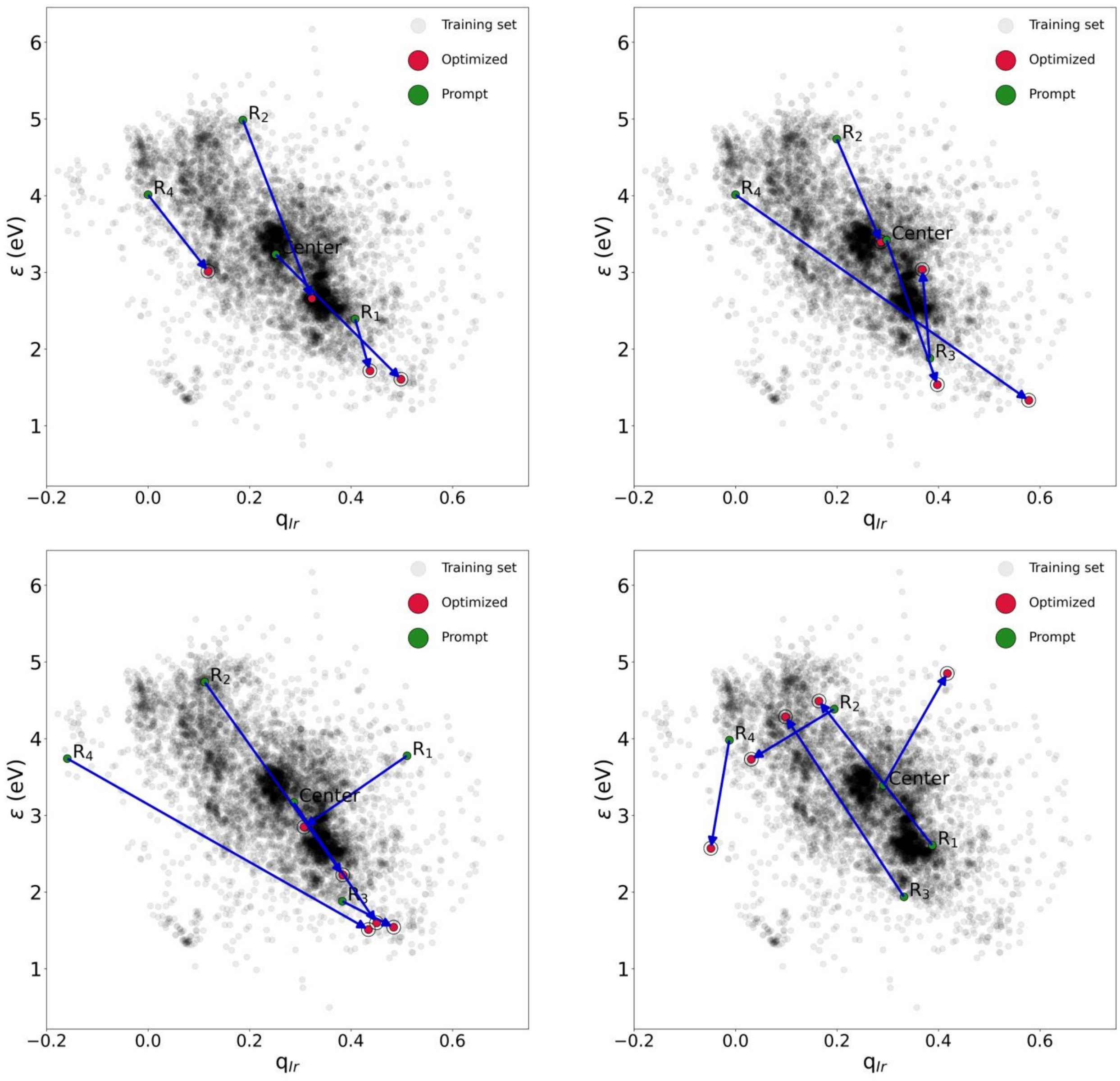

Figure S15: Conditional optimization with the κ^2 JT-VAE model in $Y = (\epsilon, q_{Ir})$ space. From top to bottom, and from left to right, clockwise: Longest trajectories in the single-objective minimization of ϵ, the single-objective maximization of q_{Ir}, the dual-objective minimization of ϵ and maximization of q_{Ir}, and the dual-objective maximization of ϵ and minimization of q_{Ir}.

S8.1 Latent space analysis

Before using the conditional JT-VAE, we inspected how the latent space is structured relative to that of the unconditional model. This was done by encoding the ligands from the training set into the latent space. Then, we encoded the latent vectors into a bidimensional space using the UMAP method.[3] Figure S16 shows the UMAP embeddings of the κ^1 ligands containing the ten most frequent coordination environments in the training dataset. The latent space appeared to be rather structured, with clusters enclosing ligands for which the coordination environment was the same. Furthermore, there seems to be a higher-level separation over these groups: 1) N-coordinating ligands (bottom-left corner of the UMAP), 2) P-coordinating ligands (bottom-right), and 3) S/C/O-coordinating (top-right). Overall, the UMAP data appears much less entangled than in the DFT space of Y = (ϵ, q_{Ir}) labels (Figure S10).

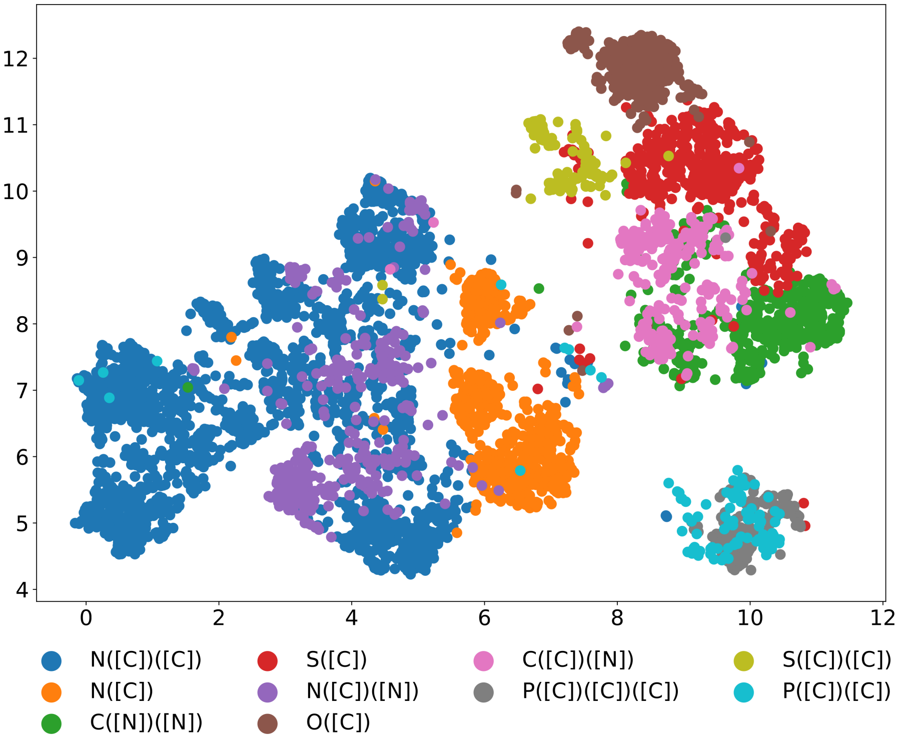

Figure S16: UMAP dimensionality reduction of the latent space for the conditional κ^1 JT-VAE model. Only points corresponding to the ten most frequent coordination environments are shown.

S8.2 Free ligand optimization trajectories in property space

Figure S17 shows two examples of free ligand optimization trajectories in property space: that of **L8**, and one trajectory that failed to follow the intended direction.

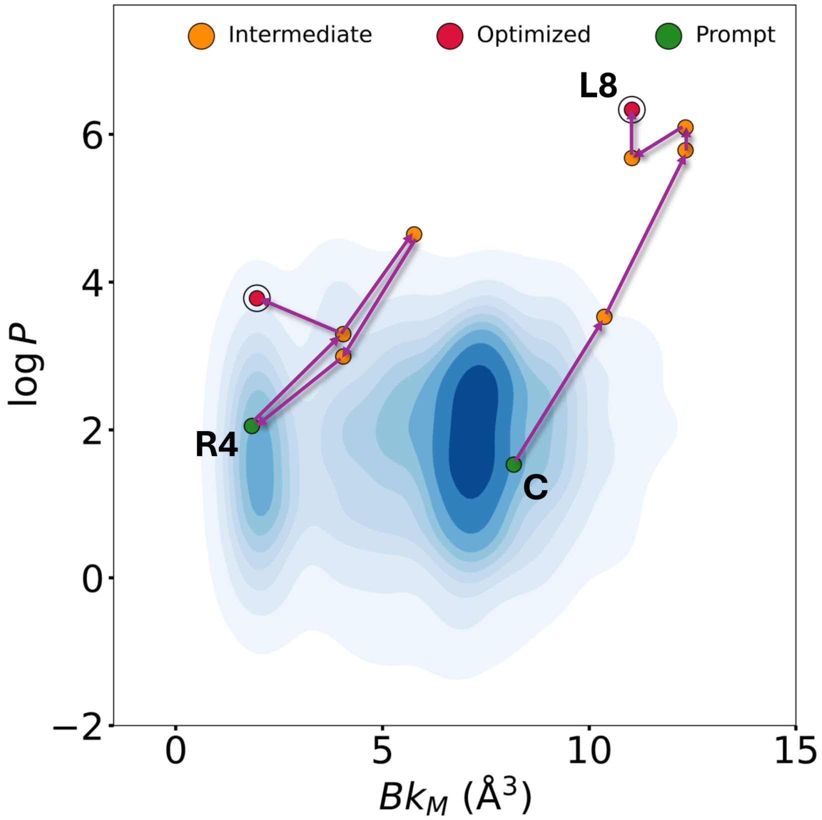

Figure S17: Trajectory of the conditional generation of ligand **L8** in property space, and example of a trajectory that failed to follow the intended direction. The contour plot in the background denotes the density of training TMCs.

S8.3 Assessment of conditional ligand generation

We assessed the results of conditional generation of ligands by way of optimizing latent space vectors with respect to target property space in Tables 2 and 3. Here we formally describe the underlying calculations as a progression of defining subsets and quantifying their relative proportions.

We consider a number of optimization trajectories $t = (i_t, f_t, o_t, r_t)$ with initial ligand i_t sampled from starting region $r_t \in \mathcal{R}$, optimized in an optimization direction $o_t \in \mathcal{D}$

and yielding some final ligand f_t. Here both optimization direction o_t and starting region r_t are expressed with respect to the property space. Then, in Tables 2 and 3 we have fractions (represented as percentages) of related subsets, for each of the starting regions $r \in \mathcal{R} = \{\text{R1}, ..., \text{R4}, \text{C}\}$. For each starting region, Tables 2 and 3 also designate the longest trajectories in terms of $o_t \in \mathcal{D} = \{\text{D1}, ..., \text{D8}\}$. Both tables provide metrics for similarity, validity, uniqueness, novelty, verification, and longest trajectory, as defined in the following sections.

S8.3.1 Similarity

We define the set of trajectories with a single common starting region r as

$$T_r = \{t : r_t = r\},$$

and define the associated subset of sufficiently similar final ligands as

$$S_r = \{t : \text{sim}(i_t, f_t) > s \wedge t \in T_r\},$$

where we take only those trajectories with starting region r where the similarity threshold between the initial and final ligands is above some threshold s, i.e., $\text{sim}(i_t, f_t) > s$. The value reported is this %:

$$a_r = \frac{|S_r|}{|T_r|} \cdot 100\%$$

using a similarity threshold of 0.20 (κ^1) or 0.15 (κ^2).

S8.3.2 Validity

We then define a validation function on final ligands such that valid(f_t) returns boolean `True`, if the expected type and number of substitution atoms are present in the encoded SMILES

of the final ligand, and the denticity-specific decoding function successfully processes the substituted metal atoms in the final ligand encoded SMILES string. From this we define the subset of valid trajectories as

$$V_r = \{t : \text{valid}(f_t) \wedge t \in S_r\}.$$

and the value reported is this %:

$$b_r = \frac{|V_r|}{|S_r|} \cdot 100\%$$

S8.3.3 Uniqueness

We define

$$\tilde{V}_r = \{f_t : t \in V_r\},$$

considering the fact that some final valid ligands may repeat across trajectories. We report the percentage of unique final valid ligands as %:

$$c_r = \frac{|\tilde{V}_r|}{|V_r|} \cdot 100\%$$

For convenience we are here adopting a notation whereby the tilde (˜) symbol indicates a set of ligands as opposed to a set of trajectories.

S8.3.4 Novelty

Taking the training dataset of ligands $\tilde{\mathcal{T}}$ as simply the set of encoded SMILES ligands used during training of the respective conditional JT-VAE model, and knowing that all sampled initial ligands are elements of the training set for all trajectories, $\forall t : i_t \in \tilde{\mathcal{T}}$, we define the set of novel final ligands for each starting region as

$$\tilde{N}_r = \{f : f \in \tilde{V}_r \wedge f \notin \tilde{\mathcal{T}}\}$$

and we report this %:

$$d_r = \frac{|\tilde{N}_r|}{|\tilde{V}_r|} \cdot 100\%$$

S8.3.5 Verification and longest trajectory

With respect to the novel final ligands $f \in \tilde{N}_r$, we again consider trajectories and define an associated set

$$N_r = \{t : f_t \in \tilde{N}_r\}.$$

and we aim at assessing the trajectories against the intended optimization direction

$$o_t \in \mathcal{D} = \{\mathrm{D1}, ..., \mathrm{D8}\}.$$

The abbreviated expression for the optimization direction (or, optimization objective) Dn given the target property space is given in Table S1.

Table S1: Optimization directions over different target property spaces.

Optimization direction name	Target property space: $(\epsilon, q_{\mathrm{Ir}})$	Target property space: $(\log P, Bk_M)$
	Optimization objective	
D1	$\max(\epsilon)$	$\max(\log P)$
D2	$\max(q_{\mathrm{Ir}})$	$\max(Bk_M)$
D3	$\min(\epsilon)$	$\min(\log P)$
D4	$\min(q_{\mathrm{Ir}})$	$\min(\log B)$
D5	$\max(\epsilon + q_{\mathrm{Ir}})$	$\max(\log P + Bk_M)$
D6	$\min(\epsilon - q_{\mathrm{Ir}})$	$\min(\log P - Bk_M)$
D7	$\min(\epsilon + q_{\mathrm{Ir}})$	$\min(\log P + Bk_M)$
D8	$\max(\epsilon - q_{\mathrm{Ir}})$	$\max(\log P - Bk_M)$

Note that we here use min and max simply to indicate whether the optimization is intended in the positive or negative direction of each property in the argument, and this is distributed to the terms of the argument modulo the sign of each term. For example,

$$\max(\log P - Bk_M) \equiv \max(\log P) \wedge \min(Bk_M)$$

indicates that $\log P$ should be maximized but Bk_M should be minimized. We can also reduce the optimization direction expression to only consider the direction component of a single property to get either

$$\begin{aligned} \text{reduce}_{p_j}(o_t) &\equiv \max(p_j) \text{ or,} \\ \text{reduce}_{p_j}(o_t) &\equiv \min(p_j). \end{aligned}$$

and continuing the previous example, we would get

$$\begin{aligned} \text{reduce}_{\log P}(\max(\log P - Bk_M)) &\equiv \max(\log P), \text{ and} \\ \text{reduce}_{Bk_M}(\max(\log P - Bk_M)) &\equiv \min(Bk_M). \end{aligned}$$

For the DFT-labeled optimizations, we next sample without replacement up to 10 trajectories (if less than 10 are available, take as many as are available) for each optimization direction o to obtain

$$X_{r,o} = \underset{\to 10}{\text{sample}}(\{t : o_t = o \wedge t \in N_r\}).$$

and then combine all the samples and take the set of final ligands:

$$\tilde{X} = \left\{ f_t : t \in \bigcup_{r,d} X_{r,d} \right\}.$$

noting that, for the optimizations performed with JT-VAE models conditioned on free ligand properties, the calculations were inexpensive and successful to an extent at which constrain-

ing the sampling to a subset of trajectories was not necessary.

We then assemble each of the final ligands in $\tilde{X}$ into homoleptic TMCs to get a set of TMC geometries, and submit them (the normally terminating subset of these TMC assembly processes) to DFT geometry optimization and labeling. We thus obtain a set of assembled and optimized homoleptic TMCs c_f of final ligands f comprising their respective geometries g_{c_f} and labels l_{c_f}:

$$\hat{C}_F = \left\{ c_f = (g_{c_f}, l_{c_f}) : \forall f \in \tilde{X} \Rightarrow (g_{c_f}, l_{c_f}) = \text{DFT}(\text{assemble}(f)) \right\}.$$

Here both the functions $(g, l) = \text{DFT}(g)$ and $g = \text{assemble}(f)$ return nothing if the process does not terminate normally when computed, hence adding no elements to the set $\hat{C}_F$ in such cases. We also stipulate that we can track the final ligands f associated with each tuple $c_f \in \hat{C}_F$. For optimizations conditioned on free ligand properties, the relevant non-DFT calculations must also terminate normally to be considered, and hence the same notation is applicable.

For each $c_f \in \hat{C}_F$ we then have some properties of interest $p_j(f)$ that we think of as being — albeit indirectly via the assembled and optimized homoleptic TMC — properties of the final ligand f. Depending on the training regimen of the conditional JT-VAE model used, either the properties of interest are the DFT labels

$$p_j(f) \in \mathcal{P}_{\text{DFT}} = \{\epsilon_{c_f}, q_{\text{Ir},c_f}\},$$

or else the free ligand descriptors

$$p_j(f) \in \mathcal{P}_{\text{free}} = \{\log P_f, Bk_{M,c_f}\}.$$

where $\log P_f$ can be calculated directly from the ligand SMILES, while Bk_{M,c_f} is calculated from its geometry. In addition, we always have the corresponding properties $p_j(i)$ for the initial ligands i.

Thus, for each trajectory $t \in N_r$, for each property p_j, we can check whether the difference between initial and final ligands goes in accordance with the intended optimization direction o_t. We define a function $\text{verify}_{p_j}(t)$ to return boolean `True` when the difference between $\Delta_j(t) = p_j(f_t) - p_j(i_t)$ has the same sign as the sign of that property in the optimization direction:

$$\text{verify}_{p_j}(t) = \begin{cases} \texttt{True}, & \text{if} \quad \text{sign}(p_j(f_t) - p_j(i_t)) = \text{sign}(o_t|p_j), \text{ or} \\ \texttt{False}, & \text{otherwise.} \end{cases}$$

where we use sign in the normal numerical way for the target properties p_j which are assumed to be real scalar values, but for the optimization direction o_t of a trajectory with respect to an individual property p_j we take

$$\text{sign}(o_t|p_j) = \begin{cases} +1, & \text{if} \quad \text{reduce}_{p_j}(o_t) \equiv \max(p_j), \text{ and} \\ -1, & \text{if} \quad \text{reduce}_{p_j}(o_t) \equiv \min(p_j). \end{cases}$$

and, thus, we can define the set of verified trajectories with a common starting region r:

$$W_r = \{t : c_{f_t} \in \hat{C}_F \wedge r_t \equiv r \wedge \forall p_j \in \mathcal{P}, \text{ verify}_{p_j}(t) \equiv \texttt{True}\}$$

The fraction of verified trajectories against all trajectories with assembled and optimized TMCs of novel final ligands is reported with this %:

$$e_r = \frac{|W_r|}{|\{t : t \in N_r \wedge c_{f_t} \in \hat{C}_F\}|} \cdot 100\%$$

For each starting region, we then find the optimization direction of the longest verified trajectory as determined by the Euclidean norm in the target property space with the result

$$o_r^* = \underset{o_t : t \in W_r}{\arg\max} \sqrt{\sum_{\forall p_j \in \mathcal{P}} (p_j(f_t) - p_j(i_t))^2}.$$

References

(1) Kneiding, H.; Nova, A.; Balcells, D. Directional multiobjective optimization of metal complexes at the billion-system scale. *Nat. Comput. Sci.* **2024**, *4*, 263–273.

(2) Jin, W.; Barzilay, R.; Jaakkola, T. Junction Tree Variational Autoencoder for Molecular Graph Generation. *International Conference on Machine Learning* **2018**, *80*, 2323–2332.

(3) McInnes, L.; Healy, J.; Saul, N.; Großberger, L. UMAP: Uniform Manifold Approximation and Projection. *J. Open Source Softw.* **2018**, *3*, 861.
